# Fragile X Messenger Ribonucleoprotein 1 (FMR1), a novel inhibitor of osteoblast/osteocyte differentiation, regulates bone formation, mass, and strength in young and aged male and female mice

**DOI:** 10.1038/s41413-023-00256-x

**Published:** 2023-05-17

**Authors:** Padmini Deosthale, Julián Balanta-Melo, Amy Creecy, Chongshan Liu, Alejandro Marcial, Laura Morales, Julita Cridlin, Sylvia Robertson, Chiebuka Okpara, David J. Sanchez, Mahdi Ayoubi, Joaquín N. Lugo, Christopher J. Hernandez, Joseph M. Wallace, Lilian I. Plotkin

**Affiliations:** 1 Department of Anatomy, Cell Biology & Physiology, Indiana University School of Medicine, Indianapolis, IN, 46202 USA; 2 Roudebush Veterans Administration Medical Center, Indianapolis, IN, 46202 USA; 3 Indiana Center for Musculoskeletal Health, Indianapolis, IN 46202 USA; 4 Universidad del Valle School of Dentistry, Cali, 760043 Colombia; 5 Department of Biomedical Engineering, Indiana University-Purdue University Indianapolis, 46202 Indianapolis, IN, 46202 USA; 6 Sibley School of Mechanical and Aerospace Engineering, Cornell University, Ithaca, NY 14853 USA; 7 Department of Materials Science and Engineering, Cornell University, Ithaca, NY 14853 USA; 8 Department of Psychology and Neuroscience, Baylor University, Waco, TX 76798 USA

**Keywords:** Bone, Metabolism

## Abstract

Fragile X Messenger Ribonucleoprotein 1 (FMR1) gene mutations lead to fragile X syndrome, cognitive disorders, and, in some individuals, scoliosis and craniofacial abnormalities. Four-month-old (mo) male mice with deletion of the *FMR1* gene exhibit a mild increase in cortical and cancellous femoral bone mass. However, consequences of absence of FMR1 in bone of young/aged male/female mice and the cellular basis of the skeletal phenotype remain unknown. We found that absence of FMR1 results in improved bone properties with higher bone mineral density in both sexes and in 2- and 9-mo mice. The cancellous bone mass is higher only in females, whereas, cortical bone mass is higher in 2- and 9-mo males, but higher in 2- and lower in 9-mo female FMR1-knockout mice. Furthermore, male bones show higher biomechanical properties at 2mo, and females at both ages. Absence of FMR1 increases osteoblast/mineralization/bone formation and osteocyte dendricity/gene expression in vivo*/*ex vivo*/*in vitro, without affecting osteoclasts in vivo*/*ex vivo. Thus, FMR1 is a novel osteoblast/osteocyte differentiation inhibitor, and its absence leads to age-, site- and sex-dependent higher bone mass/strength.

## Introduction

The diagnosis of autism spectrum disorders (ASD) has increased worldwide.^1^ In most cases the underlying cause of the disorder is not known. However, there is an association of Fragile X syndrome (FraX) and autistic spectrum behavior, which is found in 50%–65% of boys and 20% of girls with FraX.^2^ FraX has an approximate prevalence rate of 1 in 5 000–8 000 females and 1 in 4 000 in males and is a significant monogenetic cause of autism.^3^ FraX results from absence of expression of the FMR1 protein (FMRP) due to excessive trinucleotide (CGG) repeats in the Fragile X Messenger Ribonucleoprotein 1 (*FMR1*) gene,^4^ with >200 repeats leading to a what is known as full mutation, or absence of the gene product. *FMR1* is encoded in the X chromosome and therefore, males can be either *FMR1*^*y/+*^ or *FMR1*^*y/*−^, expressing or not the gene. On the other hand, 3 genotypes are possible in females, *FMR1*^*+/+*^*, FMR1*^*+/*−^, *or FMR1*^−*/*−^. This difference underlies the fact that the incidence of the condition is higher in males and that most reported studies were performed only in male mice.

To study FraX, mice with global deletion of the FMR1 gene (*Fmr1*^−/−^ mice) have been developed, which mimic the human condition that results from more than 200 trinucleotide repeats,^5,6^ and are widely used to understand the basis of FraX. These FMR1-deficient mice, similar to humans with FraX, exhibit craniofacial abnormalities.^2,7–10^ The skeletal manifestations of the condition frequently include frontal bossing (bilateral bulging of the lateral frontal bone prominences), large forehead, increased facial length, macrocephaly, and increased mandible length in males and females both in mouse models and in humans.^9,11^ In addition, a recent study showed that 4-month-old male FMR1-deficient mice exhibit increased body and muscle weight, lower adipose tissue weight, and a mild femoral bone phenotype with high cortical thickness in the femoral mid-diaphysis and cancellous bone volume fraction (BV/TV) in the distal femur, when compared to wild type controls.^12^ However, limitations of this study include the fact that the skeleton of female mice was not analyzed, and presented a rather narrow description of the skeletal phenotype of the males at 4 months of age.

In addition to the consequences at the organismal level, absence or abnormalities in FMRP result in changes in neuronal dendrite morphology. Thus, post-mortem studies in brains from individuals with FraX and mice with FMR1 deletion showed the presence of abundant long and thin dendritic spines.^13^ Interestingly, the demonstration of this phenotype depends on the method used to put dendrites in evidence, and the area of the brain studied. Other manifestations of lack of FMR1 expression in the brain include changes in dendritic spine dynamics and innervation.^14^ Yet, whether the absence of the *FMR1* gene also alters the morphology of osteocytes, which exhibit similarities with neurons at the structural and molecular levels,^15^ remained heretofore unknown.

We now report the results of an in depth analysis of the skeletal phenotype of male and female FMR1-deficient mice. We chose to examine young 2-month-old mice at the age of sexual maturation, and aged 9-month-old mice, equivalent to ~40-year-old humans,^16^ with the purpose to determine whether absence of the FMRP alters the skeleton and its cells at the setting of puberty, or in mature adult mice, before reaching middle age. We showed that absence of FMRP results overall in high bone mass and strength, likely resulting from higher osteoblast differentiation and activity in both male and female at 2- and 9-month-old mice. The improved skeletal properties likely result from accelerated osteoblast and osteocyte differentiation, without affecting osteoclast number or function.

## Results

### FMR1 deletion results in low fat and high bone mass in male mice, and high lean and bone mass in female mice

**Fig. 1:**
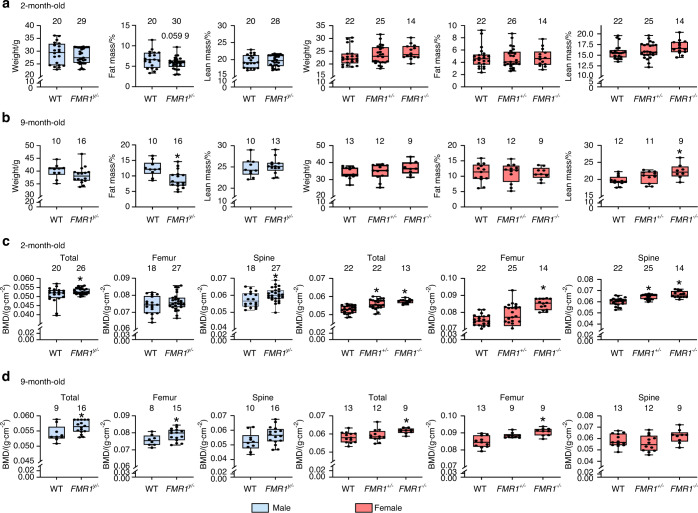
FMR1 deletion results in sex-dependent effects on fat and lean body mass in aged mice and increased bone mass in young and aged male and female FMR1-deficient mice. Animals were weighed and body composition (**a**, **b**) and BMD (**c**, **d**) were measured by Dxa/Piximus at 2 (**a**, **c**) and 9 (**b**, **d**) months of age. Data are presented as minimum to maximum box and whiskers, with box boundaries indicating 25th to 75th percentile and horizontal lines, the median, and each dot corresponding to an individual sample. *P < 0.05 vs. *FMR1*^*y/+*^ mice for males by student’s *t*-test and vs. *FMR1*^*+/+*^ by one way ANOVA for females. Detailed statistical analyses are included in Table S1 (males) and S2 (females). Numbers above the graphs indicate sample size for each measurement. BMD: bone mineral density

Mice lacking the FMR1 gene were generated in 2 different crosses, to obtain 5 different genotypes: male *FMR1*^*y/+*^ (wild type) and *FMR1*^*y/−*^ (knockout) and female *FMR1*^*+/+*^(wildtype)*, FMR1*^*+/*−^(heterozygous), *or FMR1*^−*/*−^(knockout), expressing 2, 1 or no copies of the gene. No differences in body weight were detected among the groups at 2 and 9 months of age (Fig. 1a, b). On the other hand, FMR1 deletion resulted in a tendency towards decrease in fat mass in young, and a significant decrease in aged males. For the females, the only detected difference in body composition was an increase in lean mass in 9-month-old *FMR1*^−*/*−^ mice. Deletion of FMR1 also has a sex-dependent effect on bone mineral density (BMD) (Fig. 1c, d). Thus, males showed higher whole body (total) and spine BMD at 2 months of age, and in total and femur BMD at 9 months of age. For females, both *FMR1*^*+/*−^ and *FMR1*^−*/*−^ mice exhibit higher total and spine BMD at 2 months, whereas only *FMR1*^−*/*−^ mice show higher femur BMD at 2 months, and total and femur BMD at 9 months. Thus, absence of FMR1 affects body composition in different ways depending on the sex, but results in higher bone mass in both males and females, with differences depending on the site and age analyzed.

### Absence of FMR1 leads to changes in cortical bone microstructure and strength, and in bone formation that depend on the sex- and age of the mice

**Fig. 2:**
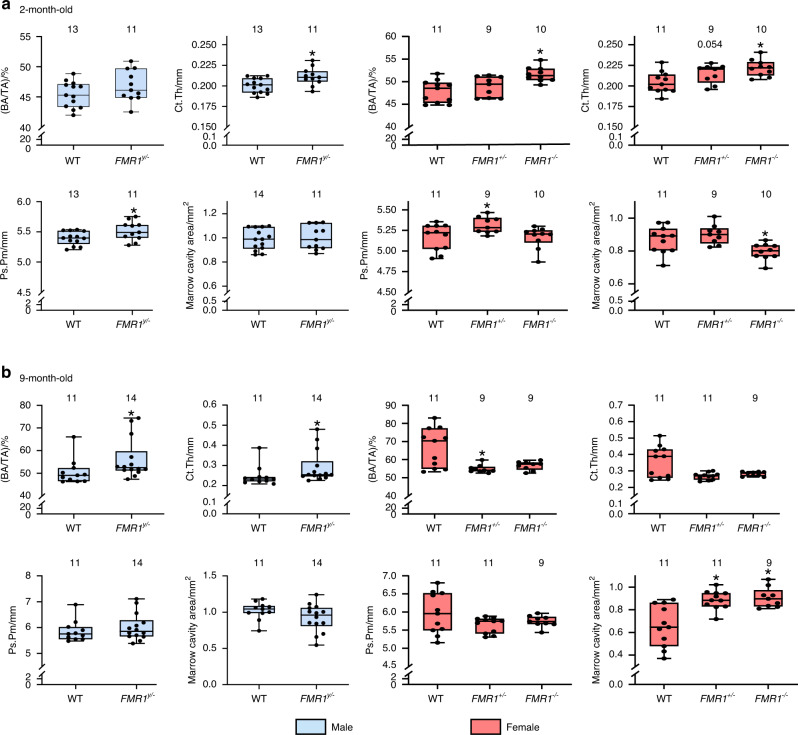
Sex and age dependent effect of FMR1 deletion in cortical bone geometry of the femoral mid-diaphysis. µCT analyses were performed at the femoral mid-diaphysis at 2 (**a**) and 9 (**b**) months of age in male and female wild type (*FMR1*^*y/+*^ and *FMR1*^*+/+*^) and deficient (*FMR1*^*y/−*^ and *FMR1*^*+/−*^ and *FMR1*^*−/−*^) mice. Data are presented as minimum to maximum box and whiskers, with box boundaries indicating 25th to 75th percentile and horizontal lines, the median, and each dot corresponding to an individual sample. *P < 0.05 vs. *FMR1*^*y/+*^ mice for males by student’s *t*-test and vs. *FMR1*^*+/+*^ by one way ANOVA for females. Detailed statistical analyses are included in Table S1 (males) and S2 (females). Numbers above the graphs indicate sample size for each measurement. BA/TA bone area/tissue area, Ct.Th cortical thickness, Ps.Pm periosteal perimeter

To further understand the consequences of FMR1 deletion in the skeleton, microstructural analyses were performed using micro-computed tomography (µCT). In males, absence of FMR1 results in higher cortical thickness, periosteal perimeter (Fig. 2a), and a tendency towards higher moment of inertia (Imax) in young mice (Fig. S1A). Moreover, FMR1 deletion resulted in increased periosteal mineral apposition rate and bone formation rate, without changes in bone formation on the endocortical surface of the femoral mid-diaphysis (Fig. S2A). By 9 months of age, there was higher bone area/tissue area and cortical thickness (Fig. 2b), but no changes in other structural parameters or in bone formation in the cortical bone surface in male mice (Fig. S1B and Fig. S2B).

The consequences of FMR1 deletion were more variable in female mice, with higher bone area and cortical area, lower marrow cavity area, and higher tissue mineral density and moment of inertia, as well as higher periosteal mineral apposition and bone formation rate in young *FMR1*^−*/*−^ mice (Fig. 2a, Fig. S1A and Fig. S2A). *FMR1*^*+/*−^ mice exhibit a tendency towards higher cortical thickness, and a surprising increase in periosteal perimeter that was not observed in *FMR1*^−*/*−^ female mice (Fig. 2a). The phenotype appears to be reversed in the older female mice, with lower bone area/tissue area only in the *FMR1*^*+/*−^ mice, and higher marrow cavity area in both heterozygous and full knockout female mice (Fig. 2b and Fig. S1B). The statistical analysis also showed a tendency in the overall effect of the deletion in periosteal mineralizing surface (*P* = 0.064, Table S2). This was accompanied by decreased endocortical bone mineralizing surface in both *FMR1*^−*/*−^ and *FMR1*^*+/*−^ mice (Fig. S2B).

FMR1 deletion also resulted in sex- and age-dependent increases in the biomechanical properties of the cortical femoral bone (Table 1), with increase in post-yield and total work in 2-month-old male and female FMR1-knockout mice, and in toughness, which only reached significance in young male mice. The effect of FMR1 deletion disappeared in the older male *FMR1*^*y/*−^ mice, but increased in the *FMR1*^−*/*−^ females, with significantly higher ultimate force, stiffness, and toughness, in addition to the differences in work observed in the younger mice, and with a slight increase in the fold change for all parameters, compared to *FMR1*^*+/+*^ mice.

**Table 1:** Biomechanical properties of femoral cortical bone assessed by 3-point bending

Age	Parameter	MALES			FEMALES			
		*FMR1*^*y/+*^	*FMR1*^*y/−*^	*FMR1*^*y/−*^*/FMR1*^*y/+*^ (fold change)	*FMR1*^*+/+*^	*FMR1*^*+/−*^	*FMR1*^*−/−*^	*FMR1*^*−/−*^*/FMR1*^*+/+*^ (fold change)
2-month-old	Yield Force /N	14.48 ± 1.94	15.58 ± 1.96	1.1	16.07 ± 3.45	17.38 ± 2.54	16.73 ± 3.58	1.0
	Ultimate Force /N	19.83 ± 1.78	21.60 ± 1.12	1.1	19.89 ± 2.76	21.59 ± 1.94	21.25 ± 2.42	1.1
	Stiffness /(N·mm^−1^)	119.03 ± 14.52	131.75 ± 10.60	1.1	132.81 ± 17.49	145.89 ± 20.36	137.96 ± 17.29	1.0
	Work to Yield /mJ	1.06 ± 0.18	1.11 ± 0.21	1.0	1.17 ± 0.37	1.24 ± 0.15	1.27 ± 0.41	1.1
	Post-yield Work /mJ	6.56 ± 1.82	**10.74** ± 4.04*	**1.6**	5.49 ± 1.70	7.10 ± 1.89	**8.40** ± **2.21**^**&**^	**1.5**
	Total Work /mJ	7.63 ± 1.91	**11.85** ± 3.92*	**1.6**	7.04 ± 1.76	8.34 ± 1.90	**9.67** ± 2.18*	**1.4**
	Yield Stress /MPa	122.32 ± 17.77	121.27 ± 23.32	1.0	141.33 ± 24.53	136.07 ± 21.82	137.54 ± 21.95	1.0
	Ultimate Stress /MPa	167.32 ± 15.04	167.57 ± 19.55	1.0	175.19 ± 16.67	169.20 ± 20.28	175.65 ± 11.10	1.0
	Modulus /GPa	6.07 ± 0.87	5.97 ± 1.04	1.0	7.08 ± 0.88	6.58 ± 1.61	6.99 ± 0.65	1.0
	Resilience /MPa	1.49 ± 0.27	1.48 ± 0.34	1.0	1.70 ± 0.48	1.71 ± 0.16	1.71 ± 0.50	1.0
	Toughness /MPa	10.73 ± 3.00	**15.68** ± 4.94*	**1.5**	10.40 ± 3.06	11.50 ± 2.42	13.24 ± 3.59	1.3
9-month-old	Yield Force /N	16.13 ± 2.43	17.15 ± 3.34	1.0	17.58 ± 3.56	18.60 ± 2.40	20.32 ± 2.74	1.2
	Ultimate Force /N	24.87 ± 3.98	26.91 ± 4.57	1.0	26.28 ± 3.26	25.71 ± 3.24	**30.46** ± 4.13*	**1.2**
	Stiffness /(N·mm^−1^)	139.76 ± 20.33	143.02 ± 27.30	1.0	154.53 ± 25.42	155.59 ± 16.89	**180.47** ± 21.22*	**1.2**
	Work to Yield /mJ	1.16 ± 0.24	1.26 ± 0.34	1.0	1.22 ± 0.34	1.37 ± 0.30	1.43 ± 0.28	1.2
	Post-yield Work /mJ	4.93 ± 2.29	5.71 ± 2.29	1.1	3.69 ± 1.05	4.05 ± 1.84	**6.05** ± 2.27*	**1.6**
	Total Work /mJ	6.09 ± 2.32	6.97 ± 2.28	1.1	4.91 ± 1.13	5.42 ± 1.63	**7.48** ± 2.16*	**1.5**
	Yield Stress /MPa	117.19 ± 19.90	118.24 ± 28.61	1.0	110.70 ± 44.64	120.44 ± 21.11	121.36 ± 14.13	1.1
	Ultimate Stress /MPa	179.05 ± 19.08	184.03 ± 32.90	1.0	158.61 ± 44.32	165.91 ± 23.45	181.69 ± 19.05	1.1
	Modulus /GPa	6.44 ± 1.08	6.17 ± 1.58	0.9	5.63 ± 2.52	6.38 ± 1.14	6.70 ± 0.90	1.2
	Resilience /MPa	1.33 ± 0.3	1.38 ± 0.39	1.0	1.32 ± 0.47	1.41 ± 0.36	1.38 ± 0.24	1.0
	Toughness /MPa	6.85 ± 2.22	7.65 ± 2.61	1.1	5.16 ± 1.30	5.49 ± 1.53	**7.24** ± 2.10*	**1.4**

males:

* = *FMR1*^*y/−*^ versus *FMR1*^*y/+*^ by two-tailed Student’s t-test

females:

* = versus *FMR1*^*+/+*^ by one way ANOVA (Multiple comparisons versus Wild Type Group, Holm-Sidak Method)

^&^ = versus *FMR1*^*+/+*^ by one way ANOVA on RANKS (Multiple comparisons versus Wild Type Group - Dunn’s Method)

Detailed statistical analyses are included in Table S5 (males) and S6 (females)

### Cancellous bone mass is only increased in female FMR1-deficient mice at both ages, and it is associated with increased osteoblast number and surface, but no changes in osteoclast-related measurements in the distal femur

**Fig. 3:**
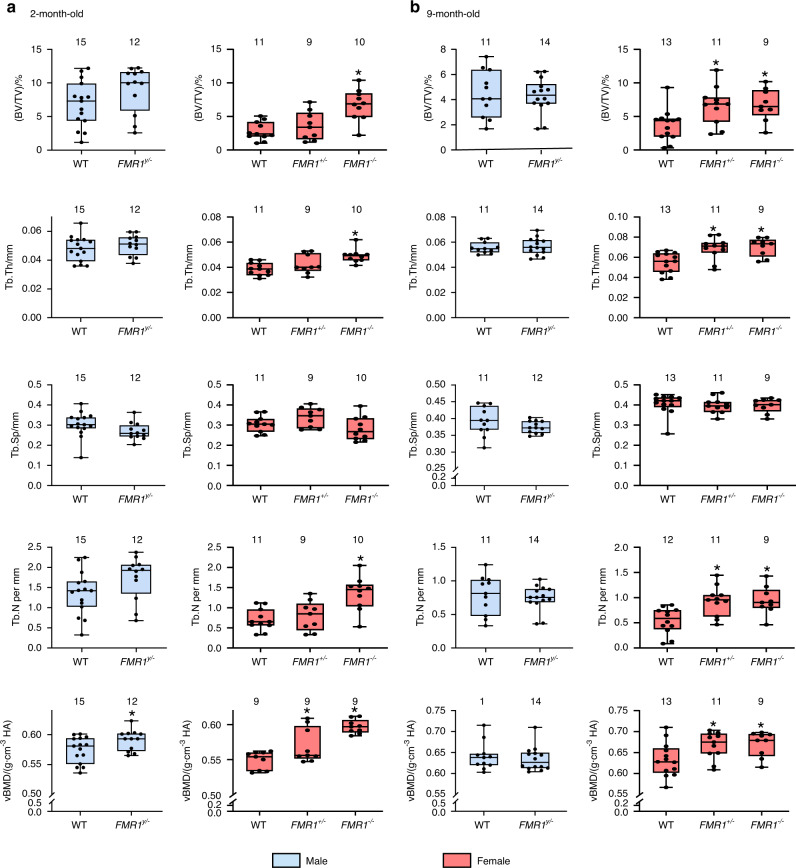
FMR1 gene deletion results in increased cancellous bone mass in the distal femur in female but not male mice at 2 and 9 months of age. Structural parameters and volumetric bone mineral density were measured by µCT in 2- (**a**) and 9- (**b**) month-old mice. Data are presented as minimum to maximum box and whiskers, with box boundaries indicating 25th to 75th percentile and horizontal lines, the median, and each dot corresponding to an individual sample. *P < 0.05 vs. *FMR1*^*y/+*^ mice for males by student’s *t*-test and vs. *FMR1*^*+/+*^ by one way ANOVA for females. Detailed statistical analyses are included in Table S1 (males) and S2 (females). Numbers above the graphs indicate sample size for each measurement. BV/TV bone volume/tissue volume, TbTh trabecular thickness, TbN trabecular number, TbSp trabecular separation, vBMD volumetric bone mineral density

We next examined the effect of FMR1 deletion in the cancellous bone of the distal femur. Overall, no changes were observed in bone isolated from male *FMR1*^*y/*−^ mice when compared to wild type littermates in cancellous bone structure at either age tested, with only a significant increase in volumetric BMD in 2-month-old mice (Fig. 3). On the other hand, FMR1 deletion resulted in higher bone volume, trabecular thickness and number and volumetric BMD in young and old *FMR1*^−*/*−^ female mice. *FMR1*^*+/*−^ mice also exhibit higher volumetric BMD at 2 months of age, and bone volume, trabecular thickness and number, as well as volumetric BMD at 9 months of age. The effect of FMR1 deletion on bone mass was associated with an overall higher bone formation parameters in young and aged *FMR1*^*+/*−^ and *FMR1*^−*/*−^ mice, with the exception of changes in mineralizing surface in 9-month-old mice (Fig. S3A, B). No changes were observed in aged male mice (Fig. S3C), whereas the effect of the deletion on bone formation was not measured in 2-month-old male mice.

The consequences of FMR1 deletion were milder in the cancellous bone of the lumbar vertebrae, with no effect in bone from male mice at either age or females at 2 months of age, except for an increase in trabecular separation that was significant for *FMR1*^*+/*−^ mice and a tendency towards higher values *P* = 0.087 - in *FMR1*^−*/*−^ mice (Fig. S4A and Table S2). In aged female mice, bone volume, trabecular thickness and number, and volumetric BMD were either higher or showed tendency towards higher values for *FMR1*^*+/*−^ and *FMR1*^−*/*−^ mice (Fig. S4B and Table S2).

**Fig. 4:**
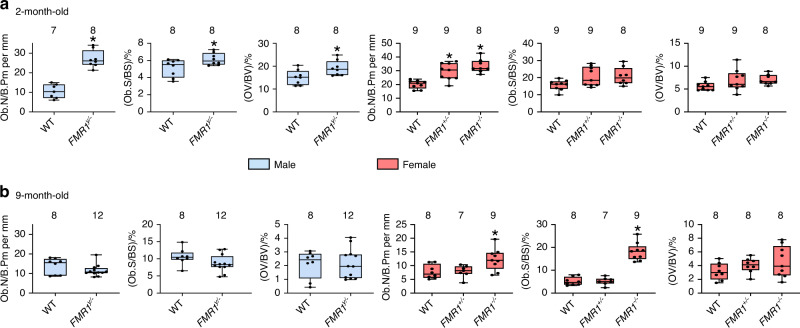
Elevated osteoblast number/surface in young male and young and old female FMR1-knockout mice. Static histomorphometric parameters were evaluated in von Kossa/McNeal- stained mineralized bone sections for osteoblast- related measurements from 2- (**a**) and 9-month-old (**b**) mice. Data are presented as minimum to maximum box and whiskers, with box boundaries indicating 25th to 75th percentile and horizontal lines, the median, and each dot corresponding to an individual sample. *P < 0.05 vs. *FMR1*^*y/+*^ mice for males by student’s *t*-test and vs. *FMR1*^*+/+*^ by one way ANOVA for females. Detailed statistical analyses are included in Table S1 (males) and S2 (females). Numbers above the graphs indicate sample size for each measurement. ObN/BPm number of osteoblasts/bone perimeter, ObS/BS osteoblast surface/bone surface, OV/BV osteoid volume/bone volume

To determine the cellular basis for the high bone mass in the FMR1-deficient mice, we performed histomorphometric analyses in cancellous bone of the distal femur. Deletion of FMR1 resulted in increased osteoblast number and surface as well as in the volume of non-mineralized bone matrix (osteoid) in 2-, but not 9-month-old male mice (Fig. 4 and Fig. S5). In females, only osteoblast number was increased in the young *FMR1*^*+/*−^ and *FMR1*^−*/*−^ mice, whereas osteoblast number and surface were higher in the full knockout aged animals (Fig. 4 and Fig. S5).

**Fig. 5:**
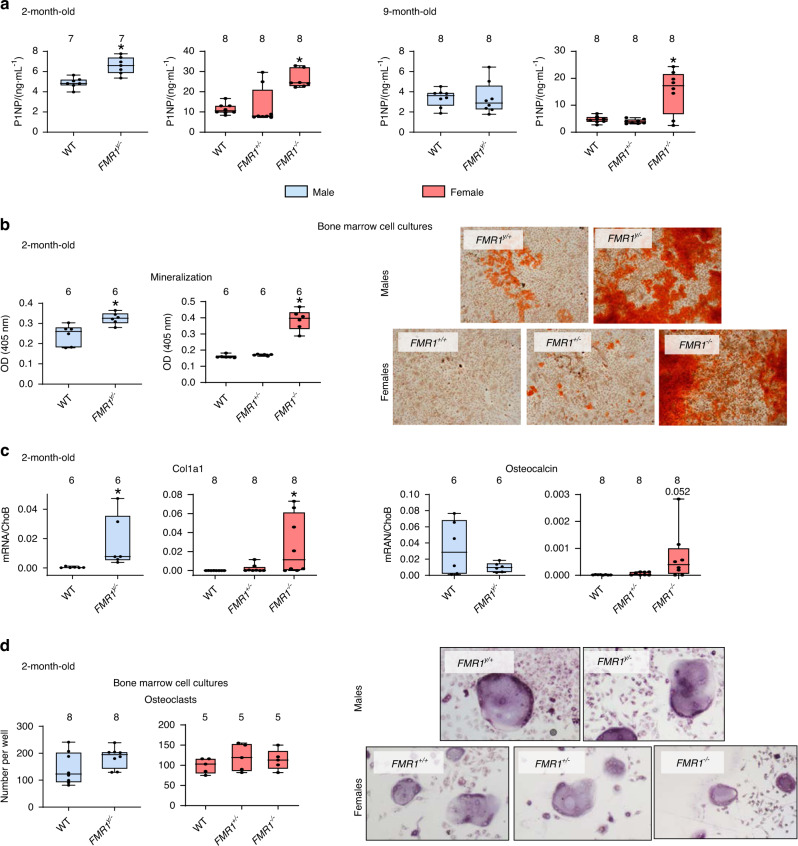
FMR1 deletion results in high bone formation in vivo and increased mineralization ex vivo. **a** The levels of the circulating marker of bone formation Procollagen 1 intact N-terminal (P1NP) were measured in serum from 2- and 9-month-old mice. **b** The level of mineralization was estimated by Alizarin red staining in bone marrow stromal cells isolated from 2-month-old mice and cultured in osteoblastogenic media for 14 days. Representative images of mineralization are shown. Scale bar corresponds to 50 µm. **c** collagen, type 1 alpha 1 (col1a1) and osteocalcin gene expression was assessed in parallel cultures of bone marrow-derived cells treated with osteoblastogenic media for 14 days. **d** The number of osteoclasts generated ex vivo from hematopoietic progenitors cultured in the presence of M-CSF/RANKL for 7 days was scored. Images are representative of osteoclast cultures. Scale corresponds to 20 µm. Box boundaries indicate 25th to 75th percentile, and the horizontal lines corresponds to the median. Vertical lines correspond to minimum to maximum values, and each dot corresponds to an individual sample. *P < 0.05 vs. *FMR1*^*y/+*^ mice for males by student’s *t*-test and vs. *FMR1*^*+/+*^ by one way ANOVA for females. Detailed statistical analyses are included in Table S1 (males) and S2 (females). Numbers above the graphs indicate sample size for each measurement. DMP1 dentin matrix protein 1, Phex phosphate regulating endopeptidase X-linked, Cx43 connexin 43, PTEN phosphatase and tensin homolog

Consistent with an overall higher osteoblast activity and bone formation in cortical and cancellous bone, the levels of the marker of bone formation P1NP, released by osteoblasts as they lay down the bone matrix, were higher in young male and female FMR1-deficient mice, and in aged female *FMR1*^−*/*−^ mice (Fig. 5a). Further, bone marrow cells isolated from 2-month-old mice and induced to differentiate in the presence of ascorbic acid and β-glycerophosphate, produce more mineral, as evidenced by the staining with Alizarin red (Fig. 5b). Moreover, these differentiated osteoblastic cells expressed higher levels of collagen1a1, the main component of the bone matrix, whereas no difference was detected in the mRNA levels for osteocalcin, an osteoblast-specific gene, although we detected a tendency towards higher levels in the cells from *FMR1*^−*/*−^ mice (Fig. 5c). On the other hand, the number of osteoclasts differentiated from bone marrow cells isolated from 2-month-old mice did not differ among groups for either male or female mice (Fig. 5d).

### FMR1 silencing leads to increased expression of genes associated with osteocyte differentiation in IDG-SW3 osteocytic cells

**Fig. 6:**
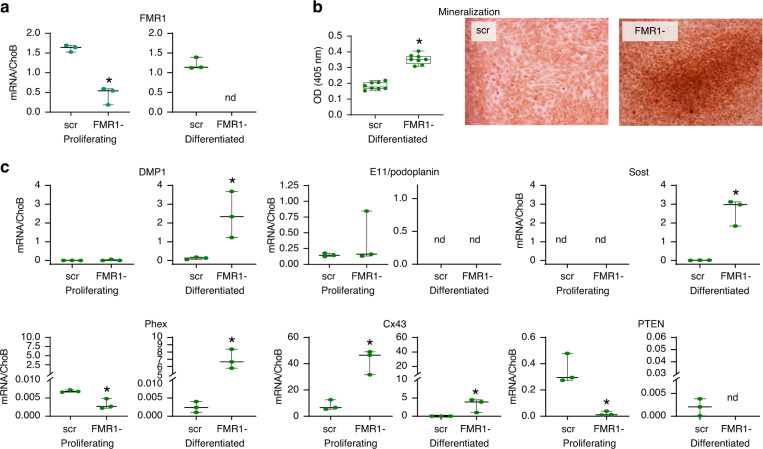
Deletion of FMR1 in IDG-SW3 osteocytic cells leads to increased mineralization and expression of osteocytic genes. **a** FMR1 gene expression level was assessed by qPCR in IDG-SW3 scrambled control (scr) and FMR1 knockout (FMR1-) cells (*n* = 3 per group). **b** Mineralization was induced in IDG-SW3 in the presence of osteoblastogenic media for 28 days (*n* = 8 per group) and was assessed by Alizarin red staining. **c** Gene expression in IDG-SW3 scr and FMR1- cells at their proliferating and differentiated stage (*n* = 3 per group). Images are representative of mineralized cultures after 28 days. Scale bar corresponds to 50 µm. Box boundaries indicate 25th to 75th percentile, and the horizontal lines corresponds to the median. Vertical lines correspond to minimum to maximum values, and each dot corresponds to an individual sample. *P < 0.05 vs. *FMR1*^*y/+*^ mice for males by student’s *t*-test and vs. *FMR1*^*+/+*^ by one way ANOVA for females. Detailed statistical analyses are included in Table S3

To determine whether deletion of FMR1 has a cell autonomous effect on osteoblastic cells, and it does not depend on an FMR1-deficient environment, we used CRISPR/Cas9 to knockdown the FMR1 gene in proliferating IDG-SW3 osteoblastic cells, which can be induced to mineralize in vitro.^17^ We demonstrated effective FMR1 deletion in both proliferating cells and in cells induced to differentiate for 28 days (Fig. 6a). And, as with the bone marrow cells isolated from the mice, deletion of FMR1 resulted in higher levels of mineralization in the knock-down cells (Fig. 6b). Consistent with previous studies, differentiated IDG-SW3 cells expressed markers of osteocytes,^17^ including DMP1, Sost and Phex, whereas the late osteoblast marker E11 decreased in the differentiated cells (Fig. 6c). FMR1 knockdown further increased the expression of osteocytic genes, In addition, FMR1 deficiency resulted in higher Cx43 and lower PTEN levels in proliferating and differentiated cells, both changes associated with reduced osteocyte apoptosis.^18,19^ Taken together, this evidence suggests that FMR1 is an inhibitor of osteocytogenesis that might also regulate cell survival.

### Reduced FMR1 expression in MLO-Y4 osteocytic cells using CRISPR/Cas9 and in osteocytes isolated from male and female FMR1-deficient mice results in increased dendricity

**Fig. 7:**
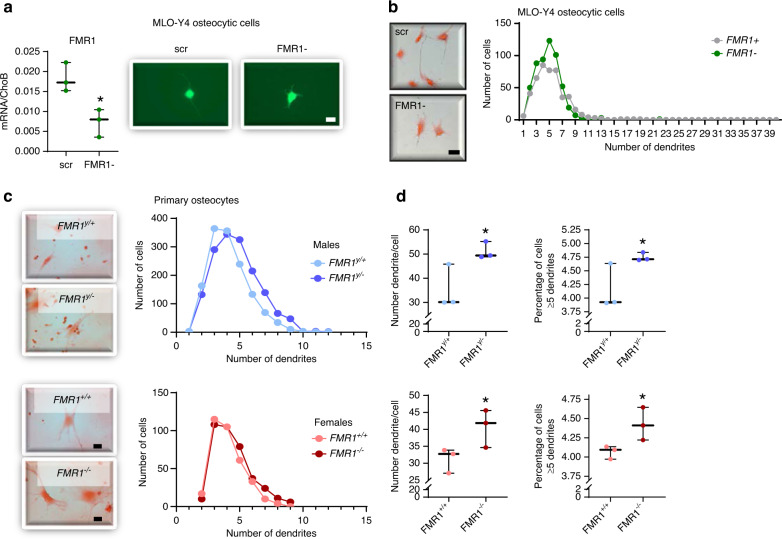
FMR1 knock down in MLO-Y4 osteocytic cells and knockout in osteocytes isolated from FMR1-deficient mice results in increased osteocyte dendricity. **a** FMR1 gene expression level was assessed by qPCR in MLO-Y4 scramble (scr) and FMR1- cells (*n* = 3 per group). Representative images show effective green fluorescent protein expression following silencing. Scale bar corresponds to 25 μm. **b** Number of cells with 5 or more dendrites in MLO-Y4 scr and FMR1- cells (*n* = 3 per group). Representative images stained with H&E are shown. Scale bar corresponds to 40 μm. **c** Distribution of dendrite number in cells isolated from wild type and FMR1-deficient male and female mice. Representative images of cells stained with H&E are shown. Scale bar corresponds to 40 µm. **d** Mean dendrite number/cell and percentage of cells with 5 or more dendrites in primary osteocytes isolated from wild type and FMR1-deficient mice. Box boundaries indicate 25th to 75th percentile, and the horizontal lines corresponds to the median. Vertical lines correspond to minimum to maximum values, and each dot corresponds to an individual sample. *P < 0.05 vs. *FMR1*^*y/+*^ mice for males by student’s *t*-test and vs. *FMR1*^*+/+*^ by one way ANOVA for females. Detailed statistical analyses are included in Table S3 (in vitro) and S4 (ex vivo)

Osteocytogenesis involves the transition from cuboidal osteoblasts into star-shaped osteocytes characterized by the presence of numerous dendrites.^20^ We then aimed to determine whether, in addition to affecting osteoblast/osteocyte gene expression, FMR1 knockdown induced changes in osteocyte dendricity. Because IDG-SW3 are cultured to confluency to induce differentiation, it is not possible to determine the morphology of isolated cells. We therefore used CRISPR/Cas9 to reduce FMR1 levels in MLO-Y4 cells, an osteocytic cell line that can be cultured in sub-confluent conditions.^21^ The reduction in FMR1 levels was verified by qPCR (Fig. 7a). Quantification of the number of dendrites/cell show a higher number of cells with more dendrites in FMR1 silenced cells compared to cells treated with scramble CRISPR/Cas9 constructs (Fig. 7b). To confirm the effect of FMR1 deletion also occurs ex vivo, osteocytes were isolated from long bones from 2-month-old wild type and FMR1 deficient male and female mice. Similar increases in the number of cells with more dendrites, with significantly higher number of dendrites/cell and percentage of cells with 5 or more dendrites were found (Fig. 7c, d).

### Male FMR1-deficient mice exhibit differences in osteocytic lacuno-canalicular network morphometry in situ

**Fig. 8:**
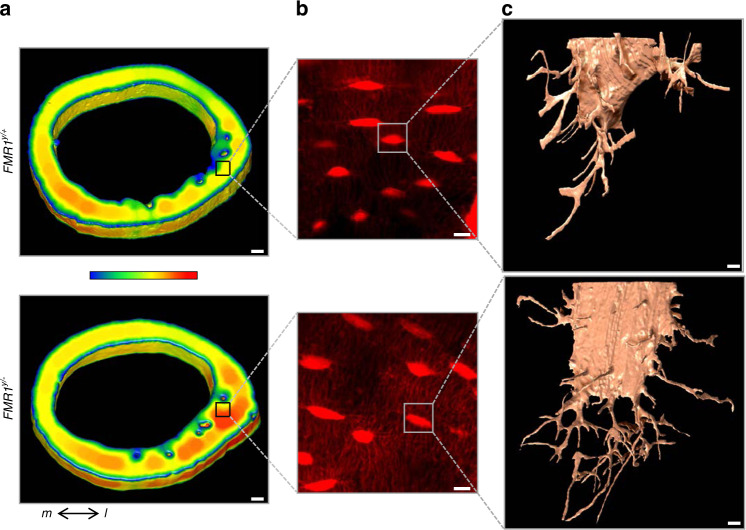
FMR1 deletion impacts microstructure of femoral cortical bone and the osteocyte lacuno-canalicular system. **a** 3D rendering of the femoral mid-diaphysis using µCT imaging, showing localized higher cortical thickness in the lateral region. µCT analysis was implemented to select a ROI (black square) for CLSM. Color bar ranges from 0 µm (blue) to 400 µm (red) thickness. **b** Z-projection of CLSM images, showing increased canaliculi density in the FMR1-deficient samples. **c** 3D meshes of lacunae and canaliculi from a randomly selected osteocyte from each ROI FIB-SEM imaging. Scale bars: (**a**) 100 µm, (**b**) 10 µm, (**c**) 1 µm. m medial, l lateral

We next examined whether the morphological changes observed ex vivo were also present in the intact bones. For this, we utilized a high-resolution 3D approach for cells embedded in the mineralized matrix,^22^ which consists in a Focused Ion Beam (FIB) combined with scanning electron microscopy (SEM), known as FIB-SEM, to describe the morphometric parameters of the lacuno-canalicular network in the femoral cortical bone of male FMR1-deficient mice and control littermates. The overall µCT evaluation of the femoral mid-diaphysis showed 5.6% higher cortical thickness in 2-month-old male *FMR1*^*y/*−^compared to FMR1^y/+^ control mice, similar to the 5.5% difference observed in the original cohort shown in Fig. 2a. Further analysis identified thicker zones on the lateral side of the cortical bone (Fig. 8a). Samples with cortical thickness matching the mean for each genotype were then selected for further analyses. We imaged the lateral region of the bone using confocal laser scanning microscopy (CLSM) and showed a clearly higher lacuna-canalicular network density (Fig. 8b). To determine the impact of the deletion of the FMR1 on the 3D arrangement of the lacuna-canalicular network in situ, a randomly selected osteocyte from each group was imaged using FIB-SEM. After artificial intelligence (AI)-assisted segmentation, each osteocyte lacunae and its associated canaliculi, defined as volume of interest or VOI, were 3D-rendered, showing remarkable differences in lacuna-canalicular network morphometry (Fig. 8c).

**Fig. 9:**
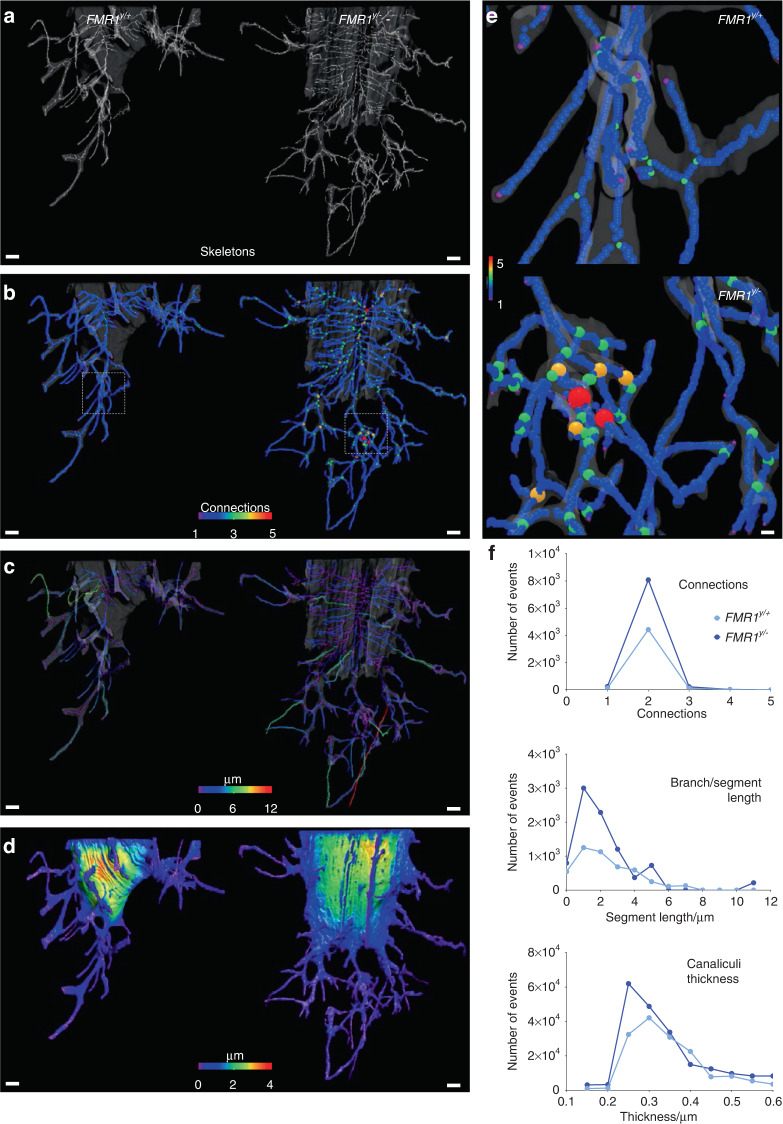
FMR1 deletion impacts the morphology of the osteocyte lacuno-canalicular system at the micro-level. **a** 3D rendering of skeletons obtained from each volume of interest (VOI) using FIB-SEM. **b** Connectivity among branches, (**c**) length of each branch/segment, and (**d**) overall thickness of the VOI were also 3D rendered. **e** Close-up view of a region from WT sample (top), where no connections involving 4–5 branches were found, contrasting the close-up view of the *FMR1*^*y/*−^ VOI (bottom). Color code indicates number of connections from 1 (blue) to 5 (red). **f** Number of events was graphed for connections among branches, length of the branches/segments, and regions thinner than 600 nm. Scale bars: (**a**–**d**) 1 µm, (**e**) 200 nm

Through further image analyses, the “skeletons” of the lacuno-canalicular network were obtained from each VOI (Fig. 9a) to identify the following morphometric parameters: the connections among branches (Fig. 9b), the length of each branch/segment (Fig. 9c), and the thickness of each branch/segment (Fig. 9d). Branches with 4–5 connections were only identified in the FMR1-deficient osteocyte, but not in the cell from the control mouse (Fig. 9e). The number of events associated with 2–3 connections among branches and branches/segments shorter than 3 µm was higher in *FMR1*^*y/*−^ compared to the WT osteocyte (Fig. 9f). In addition, considering the contribution of the canaliculi thickness to the overall thickness from the VOI, the number of events below a 600 nm thickness (the accepted maximum thickness of an osteocytic canaliculus)^23–25^ was quantified. We found that canaliculi with thickness below 350 nm were more frequent in the *FMR1*^*y/*−^ VOI. These pieces of evidence suggest that absence of FMR1 has profound effects on osteocyte morphology, increasing dendrite number and connectivity, which can be responsible, at least partially, for the phenotype of the FMR1-deficient mice.

## Discussion

We describe here for the first time the effect of reduced levels of FMR1, a gene known by its role in neurobiology,^2^ in the regulation of osteoblast/osteocyte differentiation and the control of bone mass and strength. Thus, in addition to the reported increase in bone mass in 4-month-old male mice,^12^ global FMR1 removal results in an overall higher bone mass and strength at 2 and 9 months of age in both male and female mice. The consequences of absence of the FMR1 gene at the bone tissue level are associated with increased osteoblast differentiation and osteocytogenesis, without any changes in osteoclasts, suggesting that FMR1 is a novel inhibitor of bone formation.

A few studies have examined the skeletal phenotype of patients with FraX.^26^ Individuals with FraX exhibit craniofacial characteristics such as prominent ears, a high-arched palate, and a long narrow face. In addition, they have reduced facial depth, narrow mid-facial with exaggerating ear prominence, and hypoplasticity of the nasal bone-cartilage.^9^ Their changes in bone morphology go beyond facial features. In one study approximately 50% of male FraX patients had flat feet, 57% had excessive laxity of joints, and ten of the 150 male patients had scoliosis.^7^ In general, prepubertal boys with FraX have a general overgrowth and some FraX patients have increased height and stature.^8^ These bone alterations may be more prominent in females with absence of *FMR1*. While some of the morphological changes could be consistent with increased osteoblast activity, no data so far has been reported regarding bone mineral density in individuals with FraX.

In spite of the overall effect of FMR1 deletion on bone mass and strength, we found differences in the response depending on the sex of the mice, their age, and the compartment analyzed. Thus, the effect of FMR1 deletion leads to a more lasting effect on bone biomechanical properties in female mice, with significant changes in 9-month-old mice that were not found in male littermates and with a slightly higher fold change than in the 2-month-old females. Similarly, the effects of FMR1 deletion in cancellous bone structure both in distal femur and in the lumbar vertebrae, and the circulating bone formation marker P1NP were detected in aged female, but not male, FMR1-deficient mice. On the other hand, cortical bone thickness was increased in young males and both the cortical relative bone volume and thickness were higher in the older male mice, whereas the initial increased in cortical bone parameters in 2-month-old female mice appears to be reversed in the 9-month-old animals, with significant lower and higher bone marrow cavity area, respectively, and similar tendencies for other measurements. Similar to our findings, both sex-independent and sex-specific changes in behavior were found in the same FMR1-deficient strain at 2 months of age.^27–29^ Thus, hyperactivity and reduced learning and memory capabilities were found in both male and female FMR1-deficient mice, whereas higher amounts of rearing behavior were only detected in *FMR1*^*y/*−^ male mice and higher repetitive behavior and motor coordination only found in female *FMR1*^−*/*−^ mice. Sex-specific changes in vocalization were also found in FMR1-deficient mice. However, the molecular bases for the observed sex differences are currently unknown. Further, whether the behavior differences are maintained as the animals aged, and whether the sex of the animals also play a role in the response to FMR1 deletion as the animals age remains to be determined.

A previous study from the Davidovic’s group investigated the skeletal phenotype of male FMR1-deficient mice at 4 months of age.^12^ As in the current report, these investigators reported an increase in cortical thickness and bone volume fraction, but no changes in other structural parameters in the femurs, or in bone mineral density. Yet, the increased in muscle weight observed in male mice in the published study was not reproduced in ours, since we did not detect any changes in lean mass, which is a surrogate for skeletal muscle. On the other hand, these investigators reported increase in whole body weight, as well as in femur length, which are absent in our FMR-deficient mice. Whether the different genotype result from the different ages of the mice studied, or the fact that whereas our mice are in a FBV background, the ones from the Leboucher’s study were backcrossed to C57BL/6 strain, remains unknown.

As in the manuscript by Leboucher et al.,^12^ until recently most bone studies only examined the phenotype of one sex of mice. Potentially prompted by the NIH rule to uses sex as a variable, more studies are now reporting both sexes when analyzing the skeletal phenotype of genetically modified mice.^30^ Therefore, only recent studies describe and compare the bone phenotype of males versus females. Among those, deletion of estrogen receptor α in osteoblast progenitors and hypertrophic chondrocytes cells results in a differential sex-dependent effects, with increased cortical bone mass in males and cancellous bone loss in females.^31^ We also showed sex-dependent effects of osteocytic miR-21 deletion, osteocytic and osteoclastic pannexin1 deletion, expression of a TREM2 variant associated with Alzheimer’s disease, and deletion of the connexin37 (Cx37) gene.^32–36^ Further, whereas our study analyzing the skeletal phenotype of mice expressing a truncated form of Cx43 showed decreased cancellous bone mass, but increased cortical thickness in female mice,^37^ a similar study while reproducing the phenotype of our female mice, showed that male mice with the truncated Cx43 exhibit increased periosteal and endosteal perimeters and decreased cortical thickness.^38^ However, whereas all this genetically modified mouse models resulted in opposite sex-dependent effects, or on effects only occurring in one sex but not the other, our current study shows similar consequences of FMR1 deletion in both males and females. This would suggest that FMR1 is a fundamental component of osteoblastic/osteocytic biology and therefore, its deletion results in similar phenotypic effects, although to a different extent depending on the sex of the animals.

Nevertheless, and similar to our findings on fat mass measured by Dxa/Piximus, 4-month-old male FMR1-deficient mice exhibited decreased adipose tissue weight.^12,39^ Further, the Davidovic’s group also showed that absence of the FMR1 gene enhances glucose tolerance and insulin response and lipolysis, resulting in significant changes in metabolic homeostasis.^39^ The mechanism of FMRP effect on metabolism was ascribed to the role of the protein in the regulation of mRNAs in the liver, leading to changes in the hepatic proteome. Whether the same genes/proteins are involved in the bone phenotype either directly in bone cells or due to changes in the liver, and whether sex influences the response to FMR1 deficient in the metabolome remains to be determined.

The cellular mechanism associated with the higher bone mass observed in the FMR1-deficient mice was examined in bone sections, as well as in cells derived from bone marrow from FMR1-deficient mice. Although osteoclasts express FMR1 (data not shown), its deletion did not alter osteoclast numbers in vivo or ex vivo. On the other hand, there was an increase in osteoblast-related measurements and in bone formation in vivo, as well as when bone marrow-derived osteoblast progenitors and an osteoblastic cell line were differentiated in vitro. Our data also points towards an accelerated osteoblast-osteocyte differentiation, with higher levels of osteocytic genes in the cells deficient of FMR1. Youlten et al describe the expression of FMR1 in bones, in osteocytes and, as in our study, in IDG-SW3 osteocytic cells induced to mineralized.^40^ Unfortunately, this study does not report statistical analysis, so it is not possible to determine whether there is an age-, sex- or differentiation-related effect on FMR1 levels. Another study using multiple mouse organs showed that FMR1 levels increase with aging when comparing 21- and 27-month-old mice with 1-, 3-, or 6-month-old mice in subcutaneous (SCAT), gonadal (GAT), and mesenteric adipose tissue (MAT).^41^ Of note, this study did not investigate the bone tissue transcriptome. Therefore, whether the changes in the skeleton in the absence of FMR1 or with aging depend on its level remains to be determined.

In addition, we found a consistent increase in the number of dendrites in vitro, ex vivo in cells isolated from FMR1-knockout male and female mice, and in situ in bones from male mice. It has been proposed that osteocyte dendrite regulate osteocyte function and changes in osteocyte dendricity correlated with cell survival.^15^ In addition, the integrity of the osteocyte lacuna-canaliculi system is essential for the response of bone to mechanical stimulation,^42^ and defects in osteocyte dendrites have been associated with skeletal diseases including osteoporosis, osteoarthritis and osteogenesis imperfecta, and has been proposed to mediate at least partially the consequences of glucocorticoid excess in the skeleton, and aging.^15,43^ Our evidence suggests that FMR1-deficient mice could be resistant to the effects of aging on bone mass and strength. However, we cannot definitively conclude that this is the case, since we perform the analysis before the mice reached peak bone mass, which occurs at around 4 months of age. It is therefore possible that the peak values for the measured parameters are reached at 4 months and start to decline thereafter. Future studies in which additional time points are analyzed are needed to establish whether indeed absence of FMR1 counteracts the effects of aging on the skeleton in mice. Further, future studies will determine whether FMR1 deletion also leads to resistance to other conditions associated with altered osteocyte function, such as immobilization and sex steroid deficiency.

In summary, we have shown that FMR1 deletion results in a skeletal phenotype that depends on the sex and age of the animals, as well as on the bone compartment studied. Our study reveals a novel regulator of bone formation that controls bone mass and strength and osteocyte dendrite formation.

## Materials and methods

### Animals

Mice were obtained from Jackson labs as follows: Stock 004624, Sex F,FVB.129P2-Pde6b<+> Tyr<c-ch> Fmr1<tm1Cgr>/J, Genotype: Homozygous for Fmr1<tm1Cgr>,JAXEAST:AX11: *FMR1*^*−/−*^ females Stock 004828, Sex M,FVB.129P2-Pde6b<+> Tyr<c-ch>/AntJ, Genotype: Homozygous for Tyr<c-ch>: WT males.^27^ Mice were mated following two different schemes, in order to obtain the five potential genotypes: female *FMR1*^*+/−*^ X Male *FMR1*^*y/+*^ and female *FMR1*^*+/−*^ X male *FMR1*^*y/−*^. Male *FMR1*^*y/+*^ (wild type) and *FMR1*^*y/−*^(knockout) and female *FMR1*^*+/+*^ (wild type), *FMR1*^*+/−*^ (heterozygous knockout) and *FMR1*^*−/−*^ (knockout) were analyzed at 2 and 9 months of age. Mice were maintained on regular diet and water ad libitum. All animal procedures were approved by the IUSM IACUC, and animal care and studies were carried out in accordance with institutional guidelines. Blood was collected from the mouse facial vein of after 4 h of fasting. Serum was separated using BD microcontainer tubes, aliquoted, and stored at −80 °C. The levels of PINP were measured using the RatLaps™ EIA kit (cat# AC-33F1; IDS, Scottsdale, AZ, USA) according to manufacturer’s instructions.

### DXA Piximus

Bone mineral density (BMD) and body composition were measured using a Dxa/Piximus instrument after phantom calibration, as previously published.^32^ Total (whole body, excluding the head and tail), femoral (whole femur) and spinal (lumbar vertebrae L1 to L6) BMD, as well as total lean and fat mass were measured.

### Micro-computed tomography

Femur and lumbar vertebrae (L5) were dissected, wrapped in a saline-containing gauze and kept at −20 °C until scanned. Bones were scanned using a Skyscan 1176 system (65 kV source, 0.5 mm Al filter, 0.7-degree rotation, two-image averaging and isotropic voxel of 9 µm).^32,34^ The terminology and units follow the standards for the field.^44^

### Mechanical testing

The consequences of FMR1 gene deletion on the biomechanical properties were tested using a three-point monotonic test to failure in a TA Electroforce 5500 instrument, as previously published.^45^ To normalize force-displacement date into stress-strain data, and calculate estimated material level properties, the values of c-anterior extreme fiber length, the furthest distance from the bone centroid to the surface in tension, and I_ML_, the moment of inertia about the medial-lateral axis, obtained from µCT of the mid-diaphysis were used,^46^ following standard beam bending equations, as described.^45^

### Bone histomorphometry

Animals were injected intraperitoneally with 30 mg·kg^−1^ calcein and 50 mg·kg^−1^ alizarin 7 and 2 days before sacrifice, respectively, for dynamic histomorphometric measurements.^32^ Femur were isolated at 2 and 9 months of age and fixed in 10% neutral buffered formalin and embedded in methyl-methacrylate using previously established methods. Histomorphometric analyses were performed using the Osteomeasure high resolution digital video system software (Osteometrics Inc, Decatur, GA, USA). Osteoblast and osteoclast parameters were scored in von-Kossa/McNeal- and TRAP/Toluidine blue-stained femoral bone sections, respectively. Dynamic measurements were performed on unstained femoral cortical and longitudinal sections. For the dynamic measurements in femoral cancellous bone, only five fields at the secondary spongiosa were scored. Images were taken using an Olympus BX51 fluorescent microscope and Olympus cellSense Entry 1.2(Build 7533) imaging software. The terminology and units follow the ASBMR Histomorphometry Nomenclature Committee guidance.^47^ All histological procedures were performed at the Histology and Histomorphometry Laboratory (ACBP, ICMH, Indiana CTSI) at Indiana University School of Medicine.

### Bone marrow cell culture and differentiation

To determine the effect of FMR1 deletion on cell differentiation, bone marrow cells were collected from femur and tibia of 2-month-old by cutting the ends of the bones and flushing the cells out with 10% fetal bovine serum (FBS)/1% penicillin/streptomycin (P/S)-containing α-MEM media. Bone marrow cells were cultured for 48 h, as previously published and non-adherent and adherent cells were separated.^32,48^ For osteoclast differentiation, bone marrow non-adherent cells were seeded at a density of 1.5 × 10^5^ per cm^2^ and differentiated in α-MEM containing 10% FBS, 1% P/S with 20 ng·mL^−1^ macrophage colony stimulating factor (M-CSF) and 80 ng·mL^−1^ receptor activator of NFκB ligand (RANKL).^19^ After 7 days of osteoclast differentiation, cells were fixed with 10% neutral buffered formalin and stained for TRAP using Acid Phosphatase, Leukocyte kit (Sigma Aldrich; cat# 387 A). Mature osteoclasts were identified as cells with 3 or more nuclei. Cells were counted using an inverted brightfield Leica DMI 4000 B microscope.

For osteoblastogenesis assays, adherent bone marrow cells containing osteoblastic cell precursors were collected by trypsinization and re-seeded at density 4 × 10^5^ per cm^2^ for mineralization analysis, and at 4 × 10^6^ per cm^2^ for RNA isolation. Cells were cultured in the presence of 5 mmol·L^−1^ of β-glycerolphosphate and 25 μg·mL^−1^ ascorbic acid (osteoblastogenic media) to induce osteoblast differentiation.^48^ After 14 days of culture, cells were fixed and the mineral deposited was stained using 40 mmol·L^−1^ alizarin red S (Sigma Aldrich; cat.#A5533) as previously published.^48^ The deposited stain was extracted with 1% cetylpyridinium chloride for 15 min and absorbance was measured at 405 nm^48^. Data is reported as optical density at 405 nm (OD).

For the IDG-SW3 scramble and FMR1- cells mineralization, cells were seeded at a density of 2.5 × 10^6^ per cm^2^ in 10% FBS/αMEM media containing interferon gamma. Cells were cultured at 33 °C and 5% CO_2_ for proliferation. At day 4, media was removed and 10% FBS/αMEM media containing 5 mmol·L^−1^ β-glycerophosphate and 25 μg·mL^−1^ ascorbic acid was added to induce mineralization. The cultures were maintained for 28 days at 37 °C and 8% CO2. Media was subsequently changed every 2/3 days. After 28 days, the cells were fixed, and the mineral deposited was stained using 40 mmol·L^−1^ alizarin red S (cat#A5533; Sigma Aldrich) and cells were imaged under a light microscope. Alizarin red was dissolved by adding 1% cetylpyridinium chloride for 30 min at room temperature with shaking. Absorbance was measured at 405 nm and data is reported as optical density (OD) for each well.

### FMR1 knockdown in osteoblastic/osteocytic cell lines

FMR1 expression was reduced in IDG-SW3^17^ and MLO-Y4^21^ cell lines using commercially available CRISPR/Cas9 constructs. Briefly, cells were cultured following appropriate conditions, and seeded at density of 0.052 × 10^6^ cells per cm^2^ in triplicates. After 24 h-culture, cells were exposed to a mix of 1 μg·μL^−1^ control (scramble) plasmid (cat# sc-418922; Santa Cruz Biotechnology, Inc) or FMR1 CRISPR/Cas9 plasmid (cat# sc-420392; Santa Cruz Biotechnology, Inc) or Opti-MEM serum-free media (cat# 11058021; Invitrogen), together with ViaFect Transfection Reagent (cat# E4981; Promega). Forty eight hours later, cells were trypsizined, resuspended in filtered 1% BSA/PBS and sorted using the BD ARIA GFP sorter in 96 well plates containing 20% FBS/αMEM. Each single cell clone was expanded, and 6 clones were chosen to test for gene expression. Out of them, one clone which had most effective FMR1 silencing, and its corresponding control were selected for IDG-SW3 cell mineralization and qPCR studies, and for dendrite quantification in MLO-Y4 osteocytic cells.

### Osteocyte isolation and dendrite quantification

Femora and tibiae were dissected from 2-month-old mice under aseptic conditions. After soft tissue removal, epiphyses were cut off and bones were flushed with PBS using a 27 G½ needle-syringe to remove bone marrow. Bones from two animals of same sex and genotype were pooled into one and cut into small pieces, serially digested for 25 min with collagenase solution (300 active U per mL collagenase type I-A in αMEM) followed by 5 mmol·L^−1^ EDTA in Dulbecco’s phosphate buffer with 1% BSA, pH 7.4.^49^ Between digestions, bone chips were washed 3X with Hank’s balanced salt solution. All steps were performed on rocker kept in 37 °C/ 5% CO2 incubator. After the serial digestions, bone chips were seeded in αMEM + 5% FBS and maintained in the incubator for 7 days. After 7 days, the bone chips were removed, and cells were fixed with 10% neutral buffered formalin for 10 min at RT. Cells were then stained with H&E and observed under a Leica DMI 4000 B microscope. Dendrites were quantified in fields with less than 75% confluency.

### RNA extraction and gene expression

RNA was isolated using Trizol (Invitrogen) as previously published.^32^ Reverse transcription was performed using the high-capacity cDNA transcription kit (Applied Biosystems, Invitrogen, Foster City, CA, USA). Quantitative PCR (qPCR) was performed using the Fast Taqman Universal PCR Mastermix in a Step One Plus real time PCR instrument (Applied Biosystems). Gene expression levels were normalized using the housekeeping gene ChoB. Primers and probes were available commercially (Applied Biosystems) or were designed using the Assay Design Center (Roche Applied Sciences), and catalog numbers and sequences, when available, are included in Table S7. Relative expression was calculated using ΔC_t_ comparative method.

### Focused ion beam-scanning electron microscopy (FIB-SEM)

Femora were isolated from 2-month-old male WT and *FMR1*^*y/*−^ littermates (3 per group) and fixed in neutral buffered formalin for 48 h. To identify ROIs with altered cortical thickness in the femoral mid-diaphysis, samples were scanned using a SkyScan 1172 µCT system (Bruker, Belgium), with the following parameters: 59 kV, 167 µA, 0.5 mm Al filter, integration time 885 ms, rotation step 0.7 degrees and 6 µm isotropic voxel size. A representative sample from each genotype was selected. Bones were then dehydrated using serial alcohol dilutions and stained with rhodamine6G/ethanol solution (0.417 g per 100 mL) for 72 h, with staining renewal every 24 h,^50^ embedded in methyl-methacrylate and 100 µm-thick slices were obtained. CLSM imaging was performed using an inverted TCS SP8 (Leica, Germany) microscope, on the lateral portion of the mid-diaphysis using 40X magnification. 3D datasets of 73 × 73 × 30 µm³, with a voxel size of 71 nm. Z-projections from representative ROIs were obtained from each group. Following CLSM, the FIB-SEM evaluation was performed using a Helios G4 UX (Thermo Fisher, USA) FIB system combined SEM.^51^ Serial images of 1 randomly selected osteocyte from each sample were acquired using serial slicing and viewing (40 nm voxel; 430 slices; acquisition time: 10 h/per dataset). The obtained datasets were manually pre-segmented using DRAGONFLY 2021.3 (Object Research Systems, Canada). Then, automatic segmentation of the osteocyte lacunar-canaliculi system was performed using a diffusion algorithm (Biomedisa).^52^ 3D rendering of the volume/thickness was performed using the thickness mesh tool of the DRAGONFLY software. Finally, using analytical tools from DRAGONFLY, “skeletons” from each digital sample were obtained and connectivity and length of branches/segments were quantified. Connectivity, length, and thickness are shown as histograms of frequency of events.

### Statistical analyses

Data were analyzed using SigmaPlot. All data was plotted as box and whiskers. Box boundaries indicate 25^th^ to 75^th^ percentile, with lines indicating minimum to maximum, and individual values included as dots. Male and female data were analyzed separately, and differences were evaluated either by Student’s *t-test* for male samples or by one-way ANOVA, with post-hoc analysis using Holm-Sidak method for multiple comparisons versus the wild type group for normally distributed data from female mice. For data that failed the Shapiro-Wilk normality or Brown-Forsythe Equal Variance Test tests, the Mann–Whitney Rank Sum Test and Kruskal–Wallis One Way Analysis of Variance on Ranks (Dunn’s method for multiple comparisons versus the wild type group) were used for male and female data, respectively. Significant and non-significant *P* values, as well as *t*-values and degrees of freedom for *t* test, and *P* and *F* values for one-way ANOVA analyses are included in Tables S1–6. Differences were considered significant when *P* < 0.05.

## Supplementary information

Suppl. Fig. 1

Suppl. Fig. 2

Suppl. Fig. 3

Suppl. Fig. 4

Suppl. Fig. 5

Supplementary Material
