# Supplementary figures and images for: Fragile X Messenger Ribonucleoprotein 1 (FMR1), a novel inhibitor of osteoblast/osteocyte differentiation, regulates bone formation, mass, and strength in young and aged male and female mice
Source: Bone Res. 2023 May 17;11:25. doi: 10.1038/s41413-023-00256-x (PMC10188597; doi:10.1038/s41413-023-00256-x)

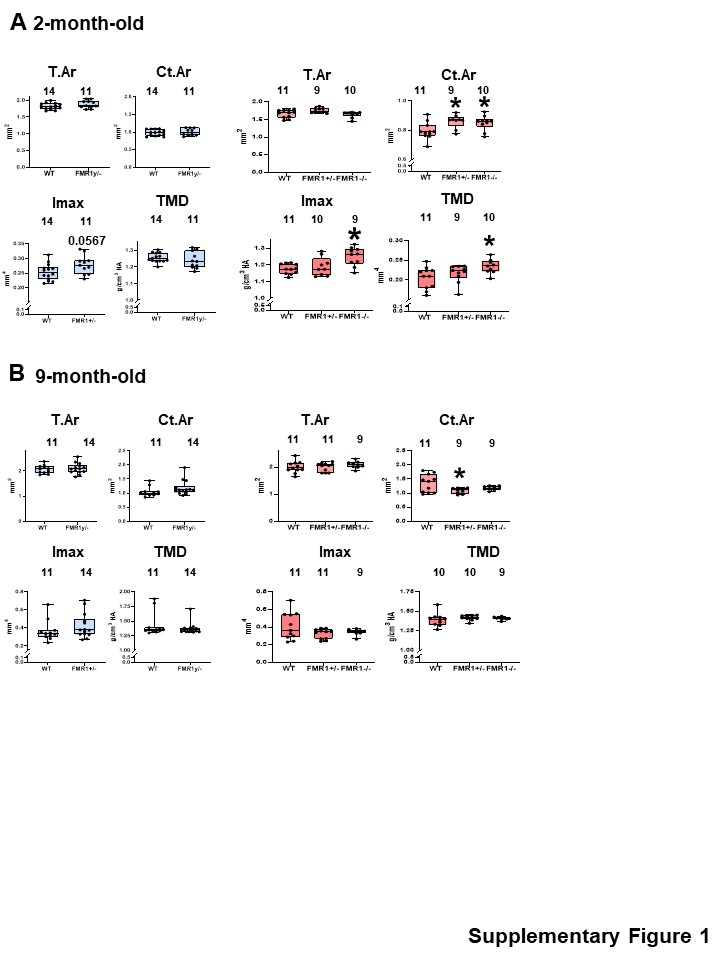

Supplement: Supplementary file 1 — Suppl. Fig. 1 [file 41413_2023_256_MOESM1_ESM.jpg]

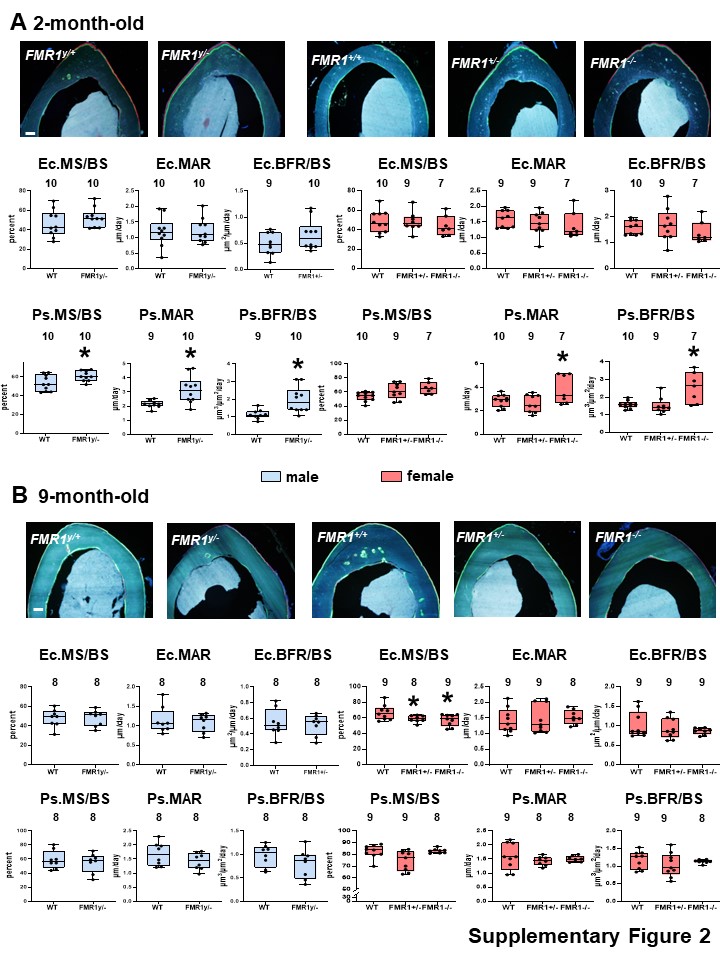

Supplement: Supplementary file 2 — Suppl. Fig. 2 [file 41413_2023_256_MOESM2_ESM.jpg]

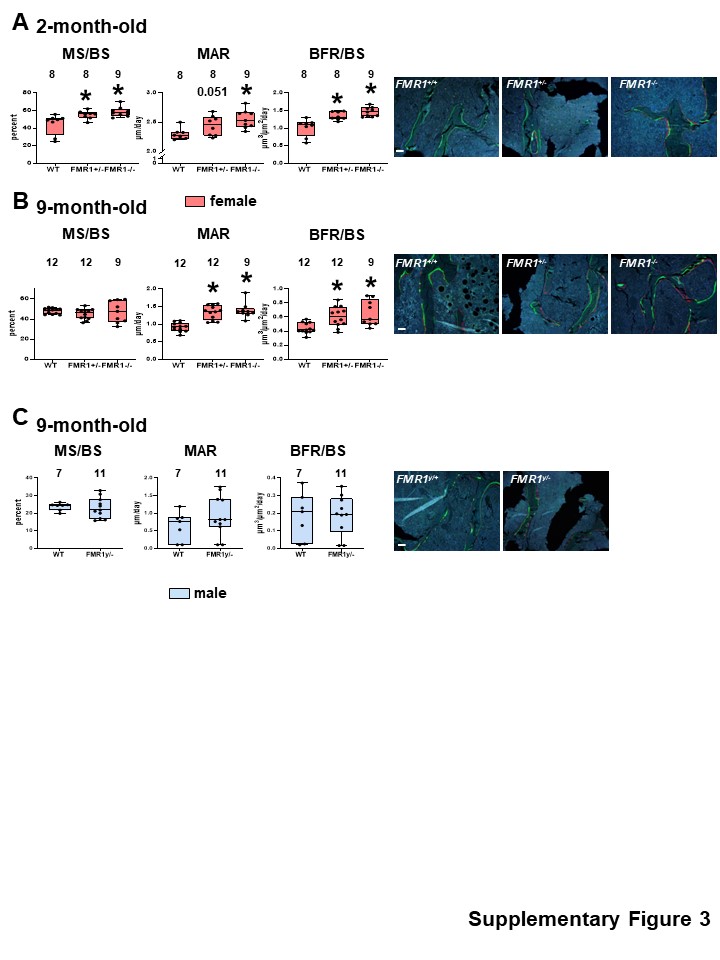

Supplement: Supplementary file 3 — Suppl. Fig. 3 [file 41413_2023_256_MOESM3_ESM.jpg]

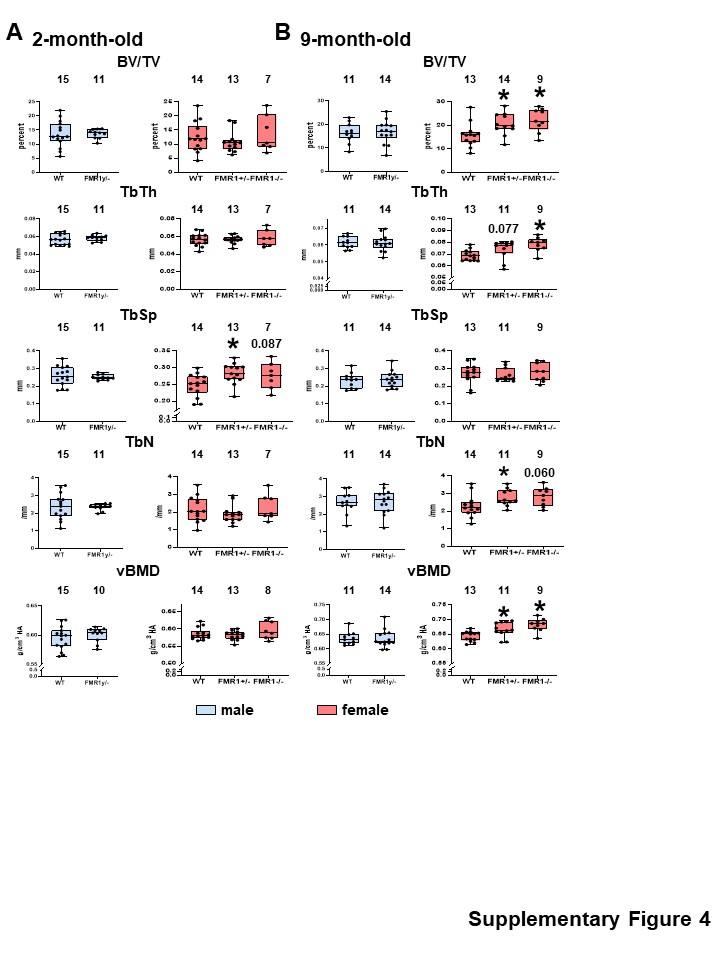

Supplement: Supplementary file 4 — Suppl. Fig. 4 [file 41413_2023_256_MOESM4_ESM.jpg]
