# Supplementary material for: Fragile X Messenger Ribonucleoprotein 1 (FMR1), a novel inhibitor of osteoblast/osteocyte differentiation, regulates bone formation, mass, and strength in young and aged male and female mice
Source: Bone Res. 2023 May 17;11:25. doi: 10.1038/s41413-023-00256-x (PMC10188597; doi:10.1038/s41413-023-00256-x)
Supplement: Supplementary file 5 — Suppl. Fig. 5 [file 41413_2023_256_MOESM5_ESM.pdf]

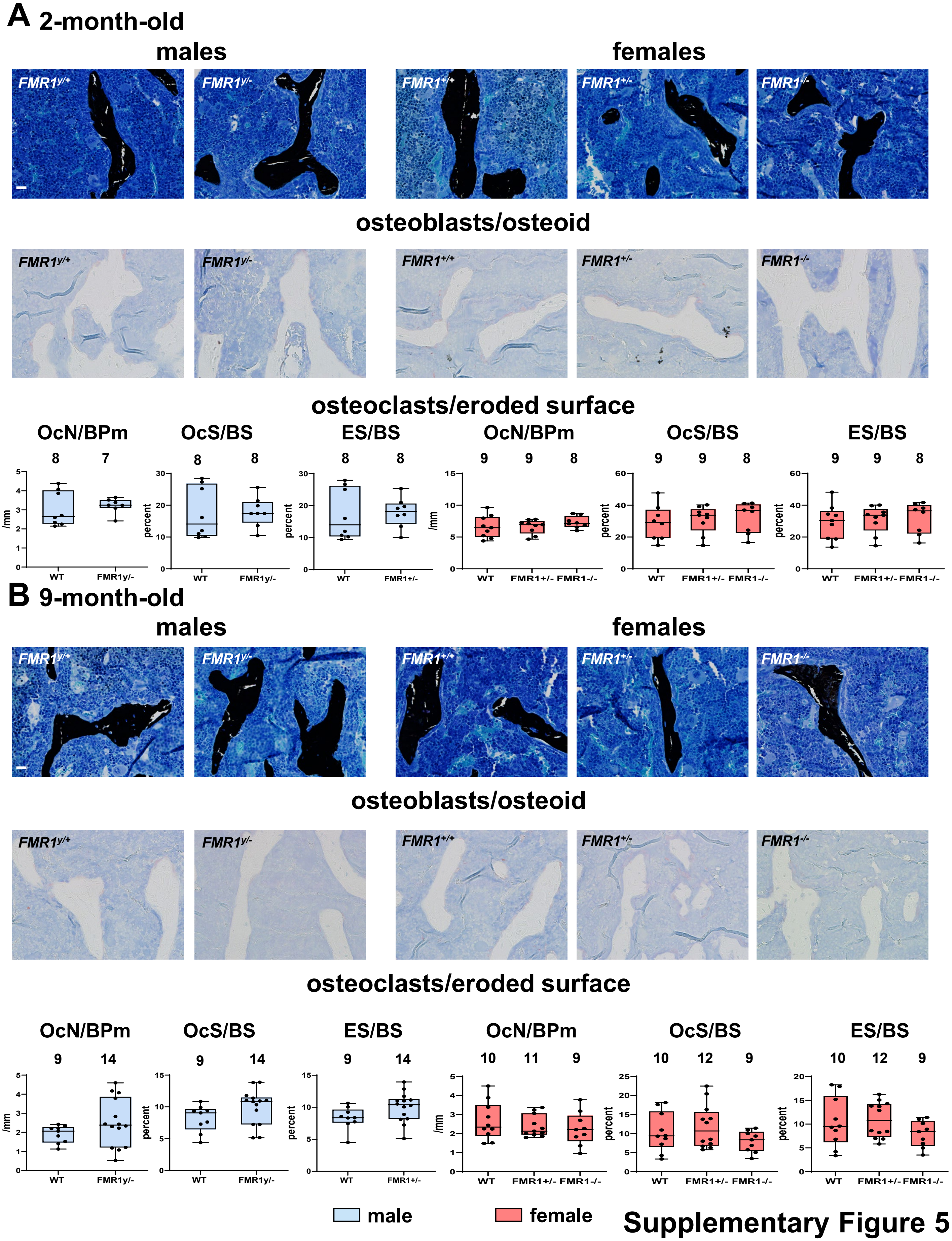

Supplementary Figure 5
