# Supplementary material for: Fragile X Messenger Ribonucleoprotein 1 (FMR1), a novel inhibitor of osteoblast/osteocyte differentiation, regulates bone formation, mass, and strength in young and aged male and female mice
Source: Bone Res. 2023 May 17;11:25. doi: 10.1038/s41413-023-00256-x (PMC10188597; doi:10.1038/s41413-023-00256-x)
Supplement: Supplementary file 6 — Supplementary Material [file 41413_2023_256_MOESM6_ESM.docx]

**Supplementary Figure Legends**

**Supplementary Figure 1. Additional analysis of the cortical bone geometry of the femoral mid-diaphysis at 2 and 9 months in male and female mice.** µCT analysis was performed at the femoral mid-diaphysis at 2 (**A**) and 9 **(B)** months of age in male and female wild type (FMR1^y/+^ and FMR1^+/+^) and deficient (FMR1^y/-^ and FMR1^+/-^ and FMR1^-/-^) mice, as in **Figure 2**. Data are presented as minimum to maximum box and whiskers, with box boundaries indicating 25^th^ to 75^th^ percentile and horizontal lines, the median, and each dot corresponding to an individual sample. *: p<0.05 vs. *FMR1^y/+^* mice for males by student’s t-test and vs. *FMR1^+/+^* by one way ANOVA for females. Detailed statistical analyses are included in **Supplementary Tables 1** (males) and **2** (females). Numbers above the graphs indicate sample size for each measurement. T.Ar: tissue area, Ct.Ar: cortical tissue area. Imax: maximum moment of inertia, TMD: tissue mineral density.

**Supplementary Figure 2. FMR1 deletion results in increased periosteal bone formation in young male and female mice but only decreased endocortical mineralizing surface in aged female mice** Dynamic histomorphometric parameters were evaluated on the periosteal and endocortical surface of the femoral mid-diaphysis in male and female mice at 2 (**A**) and 9 (**B**) months of age. Box boundaries indicate 25^th^ to 75^th^ percentile, and the horizontal lines corresponds to the median. Vertical lines indicate standard deviations, and each dot corresponds to an individual sample. *: p<0.05 vs. *FMR1^y/+^* mice for males by student’s t-test, and vs. *FMR1^+/+^* by one way ANOVA for females. Detailed statistical analyses are included in **Supplementary Tables 1** (males) and **2** (females). Numbers above the graphs indicate sample size for each measurement, and representative images for each group are shown. Scale bars corresponds to 100µm. Ps.MS/BS: periosteal mineralizing surface/bone surface, Ps.MAR: periosteal mineral apposition rate, Ps.BFR/BS: periosteal bone formation rate/bone surface, Ec.MS/BS: endocortical mineralizing surface/bone surface, Ec.MAR: endocortical mineral apposition rate, Ec.BFR/BS: endocortical bone formation rate/bone surface.

**Supplementary Figure 3. Absence of FMR1 leads to higher bone formation in young and aged female mice.** Dynamic histomorphometric analyses were performed in cancellous bone of the distal femur of female (**A and B**) and male (**C**) at the indicated ages. Box boundaries indicate 25^th^ to 75^th^ percentile, and the horizontal lines corresponds to the median. Vertical lines indicate standard deviations, and each dot corresponds to an individual sample. *: p<0.05 vs. *FMR1^y/+^* mice for males by student’s t-test, and vs. *FMR1^+/+^* by one way ANOVA for females. Detailed statistical analyses are included in **Supplementary Tables 1** (males) and **2** (females). Numbers above the graphs indicate sample size for each measurement, and representative images for each group are shown. Scale bars corresponds to 50µm. MS/BS: periosteal mineralizing surface/bone surface, MAR: periosteal mineral apposition rate, BFR/BS: periosteal bone formation rate/bone surface.

**Supplementary Figure 4. Deletion of FMR1 results in increased cancellous bone of the lumbar vertebrae only in female 9-month-old mice.** µCT analyses were performed in the 5^th^ lumbar vertebrae of 2- (**A**) and 9- (**B**) month-old male and female mice. Box boundaries indicate 25^th^ to 75^th^ percentile, and the horizontal lines corresponds to the median. Vertical lines indicate standard deviations, and each dot corresponds to an individual sample. *: p<0.05 vs. *FMR1^y/+^* mice for males by student’s t-test, and vs. *FMR1^+/+^* by one way ANOVA for females. Detailed statistical analyses are included in **Supplementary Tables 1** (males) and **2** (females). Numbers above the graphs indicate sample size for each measurement. BV/TV: bone volume/tissue volume, TbTh: trabecular thickness, TbN: trabecular number, TbSp: trabecular separation, vBMD: volumetric bone mineral density.

**Supplementary Figure 5. Osteoclast-related parameters do not change in FMR1 knockout mice at either age**.

Static histomorphometric parameters were evaluated in von Kossa/McNeal for osteoblasts (**Figure 4**) or TRAP/T. blue-stained mineralized bone sections for osteoclast-related measurements from 2- (**A**) and 9-month-old (**B**) mice. Data are presented as minimum to maximum box and whiskers, with box boundaries indicating 25^th^ to 75^th^ percentile and horizontal lines, the median, and each dot corresponding to an individual sample. *: p<0.05 vs. *FMR1^y/+^* mice for males by student’s t-test and vs. *FMR1^+/+^* by one way ANOVA for females. Detailed statistical analyses are included in **Supplementary Tables 1** (males) and **2** (females). Numbers above the graphs indicate sample size for each measurement. OcN/BPm: number of osteoclasts/bone perimeter, OcS/BS: osteoclast surface/bone surface, ES/BS: eroded surface/bone surface. Representative images for cancellous femoral bone sections of 2-month-old **(A)** and 9-month-old **(B)** mice stained for von Kossa/McNeal (top) or TRAP/T. blue (bottom) are shown. Scale bars corresponds to 20µm.

**Supplementary Table 1. Descriptive statistical analyses - males – *in vivo/ex vivo***

| **Figure/panel** | **endpoint** | **2-tailed Student’s t-test** | | | **Mann-Whitney Rank Sum Test** | | |
| --- | --- | --- | --- | --- | --- | --- | --- |
|  |  | **t** | **degrees of freedom** | **p value** | **Mann-Whitney U Statistic** | **T** | **p value** |
| **1A** | weight | - | - | - | 255.500 | 534.5 | 0.483 |
|  | fat mass | 1.927 | 48 | 0.0599 | - | - | - |
|  | lean mass | - | - | - | 275.000 | 485.000 | 0.926 |
| **1B** | weight | 1.432 | 24 | 0.1655 | - | - | - |
|  | fat mass | 3.173 | 24 | **0.0041** | - | - | - |
|  | lean mass | -0.531 | 21 | 0.601 | - | - | - |
| **1C** | total BMD | -2.117 | 44 | **0.0399** | - | - | - |
|  | femur BMD | -1.291 | 43 | 0.204 | - | - | - |
|  | spine BMD | -2.230 | 43 | **0.0311** | - | - | - |
| **1D** | total BMD | -2.279 | 23 | **0.0323** | - | - | - |
|  | femur BMD | -2.113 | 21 | **0.0467** | - | - | - |
|  | spine BMD | -1.730 | 24 | 0.0965 | - | - | - |
| **2A** | BA/TA | -1.462 | 22 | 0.158 | - | - | - |
|  | Ct.Th | -2.770 | 22 | **0.0112** | - | - | - |
|  | periosteal perimeter | -2.167 | 22 | **0.0414** | - | - | - |
|  | marrow cavity area | - | - | - | 69.000 | 151.000 | 0.661 |
| **2B** | BA/TA | - | - | - | 35.000 | 101.000 | **0.023** |
|  | Ct.Th | - | - | - | 32.000 | 98.000 | **0.014** |
|  | perimeter perimeter | - | - | - | 60.000 | 126.000 | 0.352 |
|  | marrow cavity area | 1.518 | 23 | 0.143 | - | - | - |
| **3A** | BV/TV | -1.637 | 25 | 0.114 | - | - | - |
|  | TbTh | -0.810 | 25 | 0.426 | - | - | - |
|  | TbSp | 1.636 | 25 | 0.114 | - | - | - |
|  | TbN | -1.795 | 25 | 0.0848 | - | - | - |
|  | vBMD | -2.134 | 25 | **0.0428** | - | - | - |
| **3B** | BV/TV | 0.247 | 23 | 0.807 | - | - | - |
|  | TbTh | -0.448 | 23 | 0.658 | - | - | - |
|  | TbSp | 1.280 | 21 | 0.215 | - | - | - |
|  | TbN | 0.478 | 23 | 0.637 | - | - | - |
|  | vBMD | - | - | - | 69.500 | 150.500 | 0.681 |
| **4A** | ObN/BPm | -7.899 | 13 | **2.6x10^-6^** | - | - | - |
|  | ObS/BS | -2.482 | 14 | **0.0264** | - | - | - |
|  | OV/BV | -2.341 | 14 | **0.0346** | - | - | - |
| **4B** | ObN/BPm | 1.373 | 18 | 0.187 | - | - | - |
|  | ObS/BS | 1.702 | 18 | 0.106 | - | - | - |
|  | OV/BV | -0.072 | 18 | 0.943 | - | - | - |
| **5A** | P1NP – 2 months | -4.521 | 12 | **0.0007** | - | - | - |
|  | P1NP – 9 months | 0.0319 | 14 | 0.795 | - | - | - |
| **5B** | mineralization | -3.397 | 10 | **0.00681** | - | - | - |
| **5C** | col1a1 | -2.313 | 10 | **0.0433** | - | - | - |
|  | osteocalcin | 1.735 | 10 | 0.1133 | - | - | - |
| **5D** | osteoclast number | -1.609 | 14 | 0.130 | - | - | - |
| **Suppl 1A** | T.Ar | 1.462 | 22 | 0.158 |  |  |  |
|  | Ct.Ar | 0.3987 | 23 | 0.6938 |  |  |  |
|  | Imax | -2.006 | 23 | 0.0567 | - | - | - |
|  | TMD | 0.958 | 23 | 0.348 | - | - | - |
| **Suppl 1B** | T.Ar | 0.4653 | 23 | 0.6461 |  |  |  |
|  | Ct.Ar | 1.485 | 23 | 0.1512 |  |  |  |
|  | Imax | - | - | - | 54.000 | 120.000 | 0.208 |
|  | TMD | - | - | - | 73.000 | 147.000 | 0.848 |
| **Suppl 2A** | Ec.MS/BS | -1.190 | 18 | 0.250 | - | - | - |
|  | Ec.MAR | -0.144 | 18 | 0.887 | - | - | - |
|  | Ec.BFR/BS | -1.444 | 17 | 0.167 | - | - | - |
|  | Ps.MS/BS | -2.387 | 18 | **0.0282** | - | - | - |
|  | Ps.MAR | -2.980 | 16 | **0.00885** | - | - | - |
|  | Ps.BFR/BS | -3.168 | 17 | **0.00562** | - | - | - |
| **Suppl 2B** | Ec.MS/BS | -0.0208 | 14 | 0.984 | - | - | - |
|  | Ec.MAR | 0.404 | 14 | 0.692 | - | - | - |
|  | Ec.BFR/BS | 0.319 | 14 | 0.754 | - | - | - |
|  | Ps.MS/BS | 0.623 | 14 | 0.543 | - | - | - |
|  | Ps.MAR | 1.429 | 14 | 0.175 | - | - | - |
|  | Ps.BFR/BS | 1.276 | 14 | 0.223 | - | - | - |
| **Suppl 3C** | MS/BS | - | - | - | 33.000 | 91.000 | 0.364 |
|  | MAR | - | - | - | 41.000 | 77.000 | 0.804 |
|  | BFR/BS | 0.565 | 17 | 0.796 | - | - | - |
| **Suppl 4A** | BV/TV | -0.0111 | 24 | 0.9127 | - | - | - |
|  | TbTh | -0.7688 | 24 | 0.4495 | - | - | - |
|  | TbSp | 0.3720 | 24 | 0.7132 | - | - | - |
|  | TbN | 0.09545 | 24 | 0.9247 | - | - | - |
|  | vBMD | -0.9374 | 24 | 0.3579 | - | - | - |
| **Suppl 4B** | BV/TV | -0.2194 | 23 | 0.8283 | - | - | - |
|  | TbTh | 0.04930 | 23 | 0.9611 | - | - | - |
|  | TbSp | -0.2523 | 23 | 0.8031 | - | - | - |
|  | TbN | -0.1808 | 23 | 0.8581 | - | - | - |
|  | vBMD | 0.07272 | 23 | 0.9427 | - | - | - |
| **Suppl 5A** | OcN/BPm | -0.471 | 13 | 0.646 | - | - | - |
|  | OcS/BS | -0.110 | 14 | 0.914 | - | - | - |
|  | ES/BS | -0.184 | 14 | 0.857 | - | - | - |
| **Suppl 5B** | OcN/BPm | -1.382 | 21 | 0.181 | - | - | - |
|  | OcS/BS | -1.630 | 21 | 0.118 | - | - | - |
|  | ES/BS | -1.858 | 21 | 0.0772 | - | - | - |

**Supplementary Table 2. Descriptive statistical analyses - females – *in vivo/ex vivo***

| **Figure/panel** | **endpoint** | **One Way Analysis of Variance**  **Multiple comparisons versus Wild Type Group - Holm-Sidak method** | | | **Kruskal-Wallis One Way Analysis of Variance on Ranks Multiple comparisons versus Wild Type Group - Dunn’s Method** | | |
| --- | --- | --- | --- | --- | --- | --- | --- |
|  |  | **F** | **degrees of freedom** | **p value** | **H** | **degrees of freedom** | **p value** |
| **1A** | weight | - | - | - | 3.473 | 2 | 0.176 |
|  | fat mass | - | - | - | 0.0458 | 2 | 0.977 |
|  | lean mass | 2.134 | 2 | 0.128 | - | - | - |
| **1B** | weight | 1.594 | 2 | 0.219 | - | - | - |
|  | fat mass | 0.00407 | 2 | 0.996 | - | - | - |
|  | lean mass | 5.778 | 2 | **overall: 0.008**  **FMR1^-/-^ vs. FMR1^+/+^: 0.004**  FMR1^+/-^ vs. FMR1^+/+^:0.145 | - | - | - |
| **1C** | total BMD | - | - | - | 25.807 | 2 | **overall: <0.001**  **FMR1^-/-^ vs. FMR1^+/+^: <0.001**  **FMR1^+/-^ vs. FMR1^+/+^: <0.001** |
|  | femur BMD | - | - | - | 22.224 | 2 | **overall: <0.001**  **FMR1^-/-^ vs. FMR1^+/+^: <0.001**  FMR1^+/-^ vs. FMR1^+/+^: 0.310 |
|  | spine BMD | 25.424 | 2 | **overall: <0.001**  **FMR1^-/-^ vs. FMR1^+/+^: <0.001**  **FMR1^+/-^ vs. FMR1^+/+^:<0.001** | - | - | - |
| **1D** | total BMD | 6.449 | 2 | **overall: 0.005**  **FMR1^-/-^ vs. FMR1^+/+^: 0.004**  FMR1^+/-^ vs. FMR1^+/+^: 0.749 |  |  |  |
|  | femur BMD | - | - | **-** | 13.044 | 2 | **overall: 0.001**  **FMR1^-/-^ vs. FMR1^+/+^: <0.001**  FMR1^+/-^ vs. FMR1^+/+^: 0.147 |
|  | spine BMD | 2.544 | 2 | 0.095 | - | - | - |
| **2A** | BA/TA | 8.483 | 2 | **overall: 0.001**  **FMR1^-/-^ vs. FMR1^+/+^: <0.001**  FMR1^+/-^ vs. FMR1^+/+^: 0.297 | - | - | - |
|  | Ct.Th | 6.086 | 2 | **overall: 0.007**  **FMR1^-/-^ vs. FMR1^+/+^: 0.004**  FMR1^+/-^ vs. FMR1^+/+^: 0.054 | - | - | - |
|  |  |  |  |  |  |  |  |
|  | periosteal perimeter | 3.660 | 2 | **overall: 0.039**  FMR1^-/-^ vs. FMR1^+/+^: 0.996  **FMR1^+/-^ vs. FMR1^+/+^: 0.047** | - | - | - |
|  | marrow cavity area | 6.322 | 2 | **overall: 0.006**  **FMR1^-/-^ vs. FMR1^+/+^: 0.038**  FMR1^+/-^ vs. FMR1^+/+^: 0.286 | - | - | - |
| **2B** | BA/TA | - | - | - | 8.148 | 2 | **overall: 0.017**  FMR1^-/-^ vs. FMR1^+/+^: 0.215  **FMR1^+/-^ vs. FMR1^+/+^: 0.009** |
|  | Ct.Th | - | - | - | 3.910 | 2 | 0.142 |
|  | periosteal perimeter | - | - | - | 1.732 | 2 | 0.421 |
|  | marrow cavity area | - | - | - | 11.749 | 2 | **overall: 0.003**  **FMR1^-/-^ vs. FMR1^+/+^: 0.007**  **FMR1^+/-^ vs. FMR1^+/+^: 0.006** |
| **3A** | BV/TV | 11.466 | 2 | **overall: <0.001**  **FMR1^-/-^ vs. FMR1^+/+^: <0.001**  FMR1^+/-^ vs. FMR1^+/+^: 0.379 | - | - | - |
|  | TbTh | 8.092 | 2 | **overall: 0.002**  **FMR1^-/-^ vs. FMR1^+/+^: <0.001**  FMR1^+/-^ vs. FMR1^+/+^: 0.158 | - | - | - |
|  | TbSp | 3.134 | 2 | 0.060 | - | - | - |
|  | TbN | 10.149 | 2 | **overall: <0.001**  **FMR1^-/-^ vs. FMR1^+/+^: <0.001**  FMR1^+/-^ vs. FMR1^+/+^: 0.536 | - | - | - |
|  | vBMD | 17.931 | 2 | **overall: <0.001**  **FMR1^-/-^ vs. FMR1^+/+^: <0.001**  **FMR1^+/-^ vs. FMR1^+/+^: 0.017** | - | - | - |
| **3B** | BV/TV | 5.530 | 2 | **overall: 0.009**  **FMR1^-/-^ vs. FMR1^+/+^: 0.009**  **FMR1^+/-^ vs. FMR1^+/+^: 0.016** | - | - | - |
|  | TbTh | - | - | - | 11.812 | 2 | **overall: 0.003**  **FMR1^-/-^ vs. FMR1^+/+^: 0.006**  **FMR1^+/-^ vs. FMR1^+/+^: 0.009** |
|  | TbSp | - | - | - | 1.313 | 2 | 0.519 |
|  | TbN | 7.527 | 2 | **overall: 0.002**  **FMR1^-/-^ vs. FMR1^+/+^: 0.003**  **FMR1^+/-^ vs. FMR1^+/+^: 0.005** | - | - | - |
|  | vBMD | 4.036 | 2 | **overall: 0.028**  **FMR1^-/-^ vs. FMR1^+/+^: 0.040**  **FMR1^+/-^ vs. FMR1^+/+^: 0.026** | - | - | - |
| **4A** | ObN/BPm | 16.778 | 2 | **overall: <0.001**  **FMR1^-/-^ vs. FMR1^+/+^: <0.001**  **FMR1^+/-^ vs. FMR1^+/+^: <0.001** | - | - | - |
|  | ObS/BS | 3.371 | 2 | 0.052 | - | - | - |
|  | OV/BV | 2.048 | 2 | 0.152 | - | - | - |
| **4B** | ObN/BPm | 5.888 | 2 | **overall: 0.009**  **FMR1^-/-^ vs. FMR1^+/+^: 0.013**  FMR1^+/-^ vs. FMR1^+/+^: 0.906 | - | - | - |
|  | ObS/BS | 62.128 | 2 | **overall: <0.001**  **FMR1^-/-^ vs. FMR1^+/+^: <0.001**  FMR1^+/-^ vs. FMR1^+/+^: 0.992 | - | - | - |
|  | OV/BV |  |  |  | 2.135 | 2 | 0.344 |
| **5A** | P1NP – 2 months |  |  |  | 9.995 | 2 | **overall: 0.007**  **FMR1^-/-^ vs. FMR1^+/+^:** **0.039**  FMR1^+/-^ vs. FMR1^+/+^: 0.922 |
|  | P1NP – 9 months |  |  |  | 10.771 | 2 | **overall: 0.005**  **FMR1^-/-^ vs. FMR1^+/+^: 0.001**  FMR1^+/-^ vs. FMR1^+/+^: 0.973 |
| **5B** | mineralization | - | - | - | 13.066 | 2 | **overall: 0.001**  **FMR1^-/-^ vs. FMR1^+/+^: <0.001**  FMR1^+/-^ vs. FMR1^+/+^: 0.389 |
| **5C** | col1a1 | - | - | - | 10.655 | 2 | **overall: 0.005**  **FMR1^-/-^ vs. FMR1^+/+^: 0.003**  FMR1^+/-^ vs. FMR1^+/+^: 0.074 |
|  | osteocalcin | - | - | - | 5.915 | 2 | 0.052 |
| **5D** | osteoclast number | 0.775 | 2 | 0.482 | - | - | - |
| **Suppl 1A** | T.Ar | 4.081 | 27 | **overall: 0.0283**  FMR1^-/-^ vs. FMR1^+/+^: 0.6881  FMR1^+/-^ vs. FMR1^+/+^: 0.0556 | - | - | - |
|  | Ct.Ar | 4.614 | 27 | **overall: 0.0189**  **FMR1^-/-^ vs. FMR1^+/+^: 0.0235**  **FMR1^+/-^ vs. FMR1^+/+^: 0.0204** | - | - | - |
|  | Imax | 3.681 | 2 | **overall: 0.039**  **FMR1^-/-^ vs. FMR1^+/+^: 0.023**  FMR1^+/-^ vs. FMR1^+/+^: 0.306 | - | - | - |
|  | TMD | 7.069 | 2 | **overall: 0.003**  FMR1^-/-^ vs. FMR1^+/+^: 0.0894  **FMR1^+/-^ vs. FMR1^+/+^: 0.0204** | - | - | - |
| **Suppl 1B** | T.Ar | 0.7655 | 26 | overall: 0.4746 | - | - | - |
|  | Ct.Ar | 4.002 | 26 | **overall: 0.035**  FMR1^-/-^ vs. FMR1^+/+^: 0.0894  **FMR1^+/-^ vs. FMR1^+/+^: 0.0204** | - | - | - |
|  | Imax | - | - | - | - | - | - |
|  | TMD | - | - | - | 2.421 | 2 | 0.298 |
| **Suppl 2A** | Ec.MS/BS | 0.409 | 2 | 0.669 | - | - | - |
|  | Ec.MAR | 0.726 | 2 | 0.495 | - | - | - |
|  | Ec.BFR/BS | 0.879 | 2 | 0.429 | - | - | - |
|  | Ps.MS/BS | 2.810 | 2 | 0.137 | - | - | - |
|  | Ps.MAR | 4.215 | 2 | **overall: 0.028**  **FMR1^-/-^ vs. FMR1^+/+^: 0.035**  FMR1^+/-^ vs. FMR1^+/+^: 0.923 | - | - | - |
|  | Ps.BFR/BS | 4.215 | 2 | **overall: 0.028**  **FMR1^-/-^ vs. FMR1^+/+^: 0.035**  FMR1^+/-^ vs. FMR1^+/+^: 0.923 | - | - | - |
| **Suppl 2B** | Ec.MS/BS | 3.935 | 2 | **overall: 0.034**  **FMR1^-/-^ vs. FMR1^+/+^: 0.029**  **FMR1^+/-^ vs. FMR1^+/+^: 0.045** | - | - | - |
|  | Ec.MAR | 0.185 | 2 | 0.832 |  |  |  |
|  | Ec.BFR/BS | - | - | - | 0.160 | 2 | 0.923 |
|  | Ps.MS/BS | - | - | - | 5.498 | 2 | 0.064 |
|  | Ps.MAR | - | - | - | 0.962 | 2 | 0.618 |
|  | Ps.BFR/BS | - | - | - | 0.748 | 2 | 0.688 |
| **Suppl 3A** | MS/BS | 7.166 | 2 | **overall: 0.004**  **FMR1^-/-^ vs. FMR1^+/+^: 0.003**  **FMR1^+/-^ vs. FMR1^+/+^: 0.013** | - | - | - |
|  | MAR | 6.880 | 2 | **overall: 0.005**  **FMR1^-/-^ vs. FMR1^+/+^: 0.002**  FMR1^+/-^ vs. FMR1^+/+^: 0.051 | - | - | - |
|  | BFR/BS | 14.702 | 2 | **overall: <0.001**  **FMR1^-/-^ vs. FMR1^+/+^: <0.001**  **FMR1^+/-^ vs. FMR1^+/+^: 0.001** | - | - | - |
| **Suppl 3B** | MS/BS |  |  |  | 1.404 | 2 | 0.496 |
|  | MAR | 22.800 | 2 | **overall: <0.001**  **FMR1^-/-^ vs. FMR1^+/+^: <0.001**  **FMR1^+/-^ vs. FMR1^+/+^: <0.001** | - | - | - |
|  | BFR/BS | - | - | - | 10.797 | 2 | **overall: 0.005**  **FMR1-/- vs. FMR1+/+: 0.007**  **FMR1+/- vs. FMR1+/+:0.015** |
| **Suppl 4A** | BV/TV | - | - | - | 1.172 | 2 | 0.557 |
|  | TbTh | 0.119 | 2 | 0.889 | - | - | - |
|  | TbSp | 3.802 | 2 | **overall: 0.033**  FMR1^-/-^ vs. FMR1^+/+^: 0.087  **FMR1^+/-^ vs. FMR1^+/+^: 0.025** | - | - | - |
|  | TbN | 1.046 | 2 | 0.363 | - | - | - |
|  | vBMD | 1.741 | 2 | 0.192 | - | - | - |
| **Suppl 4B** | BV/TV | 5.237 | 2 | **overall: 0.011**  **FMR1^-/-^ vs. FMR1^+/+^: 0.010**  **FMR1^+/-^ vs. FMR1^+/+^: 0.026** | - | - | - |
|  | TbTh | - | - | - | 11.177 | 2 | **overall: 0.004**  **FMR1^-/-^ vs. FMR1^+/+^: 0.002**  FMR1^+/-^ vs. FMR1^+/+^: 0.077 |
|  | TbSp | 0.232 | 2 | 0.794 | - | - | - |
|  | TbN | 3.345 | 2 | **overall: 0.049**  FMR1^-/-^ vs. FMR1^+/+^: 0.060  **FMR1^+/-^ vs. FMR1^+/+^: 0.045** | - | - | - |
|  | vBMD | 8.918 | 2 | **overall: <0.001**  **FMR1^-/-^ vs. FMR1^+/+^: <0.001**  **FMR1^+/-^ vs. FMR1^+/+^: 0.014** | - | - | - |
| **Suppl 5A** | OcN/BPm | 0.759 | 2 | 0.479 | - | - | - |
|  | OcS/BS | 0.212 | 2 | 0.810 | - | - | - |
|  | ES/BS | 0.148 | 2 | 0.863 | - | - | - |
| **Suppl 5B** | OcN/BPm | 0.612 | 2 | 0.550 | - | - | - |
|  | OcS/BS | 1.721 | 2 | 0.197 | - | - | - |
|  | ES/BS | 1.550 | 2 | 0.230 | - | - | - |

**Supplementary Table 3. Descriptive statistical analyses – *in vitro* studies**

| **Figure/panel** | **endpoint** | | **2-tailed Student’s t-test** | | | |
| --- | --- | --- | --- | --- | --- | --- |
|  |  |  | **t-value** | **degrees of freedom** | | **p value** |
| **6A** | | FMR1 – proliferating | 8.583 | 4 | | **0.0010** |
|  |  | FMR1 – differentiated | N/A | | | |
|  |  | mineralization | -12.274 | 14 | | **7x10^-9^** |
| **6B** | | DMP1 – proliferating | -0.897 | 4 | | 0.4204 |
|  |  | DMP1 - differentiated | -3.225 | 4 | | **0.0321** |
|  |  | E11 - proliferating | -0.9912 | 4 | | 0.3777 |
|  |  | E11 - differentiated | N/A | | | |
|  |  | Sost - proliferating | N/A | | | |
|  |  | Sost - differentiated | -6.485 | 4 | | **0.0029** |
|  |  | Phex- proliferating | 4.450 | 4 | | **0.0112** |
|  |  | Phex – differentiated | -9.076 | 4 | | **0.0008** |
|  |  | Cx43 – proliferating | -5.815 | 4 | | **0.0044** |
|  |  | Cx43 – differentiated | -2.896 | 4 | | **0.0443** |
|  |  | PTEN - proliferating | 5.023 | 4 | | **0.0074** |
|  |  | PTEN - differentiated | N/A | | | |
| **7A** | | FMR1 | 3.753 | 4 | **0.0199** | |

**Supplementary Table 4. Descriptive statistical analyses – *ex vivo – osteocyte cultures***

| **Figure/panel** |  | **endpoint** | **2-tailed Student’s t-test** | | |
| --- | --- | --- | --- | --- | --- |
|  |  |  | **t-value** | **degrees of freedom** | **p value** |
| **7D** | **males** | mean dendrite #/cell | -2.430 | 4 | **0.0360** |
|  |  | cells with ≥5 dendrites | -2.810 | 4 | **0.0242** |
|  | **females** | mean dendrite #/cell | -2.720 | 4 | **0.0265** |
|  |  | cells with ≥5 dendrites | -2.464 | 4 | **0.0347** |

**Supplementary Table 5. Descriptive statistical analyses – males – mechanical testing**

| **age** | **endpoint** | **2-tailed Student’s t-test** | | | **Mann-Whitney Rank Sum Test** | | |
| --- | --- | --- | --- | --- | --- | --- | --- |
|  |  | **t** | **degrees of freedom** | **p value** | **Mann-Whitney U Statistic** | **T** | **p value** |
| **2-month-old** | Yield Force (N) | -0.988 | 11 | 0.344 | - | - | - |
|  | Ultimate Force (N) | -1.980 | 11 | 0.0733 | - | - | - |
|  | Stiffness (N/mm) | -1.686 | 11 | 0.120 | - | - | - |
|  | Work to Yield (mJ) | -0.408 | 11 | 0.691 | - | - | - |
|  | Post-yield Work (mJ) | -2.583 | 11 | **0.0255** | - | - | - |
|  | Total Work (mJ) | -2.632 | 11 | **0.0233** | - | - | - |
|  | Yield Stress (MPa) | 0.0921 | 11 | 0.928 | - | - | - |
|  | Ultimate Stress (MPa) | -0.0259 | 11 | 0.980 | - | - | - |
|  | Modulus (GPa) | 0.196 | 11 | 0.848 | - | - | - |
|  | Resilience (MPa) | 0.0452 | 11 | 0.965 | - | - | - |
|  | Toughness (MPa) | -2.275 | 11 | **0.0439** | - | - | - |
| **9-month-old** | Yield Force (N) | -0.845 | 23 | 0.407 | - | - | - |
|  | Ultimate Force (N) | -1.173 | 23 | 0.253 | - | - | - |
|  | Stiffness (N/mm) | -0.331 | 23 | 0.744 | - | - | - |
|  | Work to Yield (mJ) | -0.823 | 23 | 0.419 | - | - | - |
|  | Post-yield Work (mJ) | -0.841 | 23 | 0.409 | - | - | - |
|  | Total Work (mJ) | -0.947 | 23 | 0.354 | - | - | - |
|  | Yield Stress (MPa) | -0.104 | 23 | 0.918 | - | - | - |
|  | Ultimate Stress (MPa) | -0.445 | 23 | 0.661 | - | - | - |
|  | Modulus (GPa) | 0.488 | 23 | 0.630 | - | - | - |
|  | Resilience (MPa) | -0.359 | 23 | 0.723 | - | - | - |
|  | Toughness (MPa) | -0.809 | 23 | 0.427 | - | - | - |

**Supplementary Table 6. Descriptive statistical analyses – females – mechanical testing**

| **age** | **endpoint** | **One Way Analysis of Variance**  **Multiple comparisons versus Wild Type Group - Holm-Sidak method** | | | **Kruskal-Wallis One Way Analysis of Variance on Ranks Multiple comparisons versus Wild Type Group**  **- Dunn’s Method** | | |
| --- | --- | --- | --- | --- | --- | --- | --- |
|  |  | **F** | **degrees of freedom** | **p value** | **H** | **degrees of freedom** | **p value** |
| **2-month-old** | Yield Force (N) | 0.292 | 2 | 0.750 | - | - | - |
|  | Ultimate Force (N) | 1.057 | 2 | 0.366 | - | - | - |
|  | Stiffness (N/mm) | 0.932 | 2 | 0.410 | - | - | - |
|  | Work to Yield (mJ) |  |  |  | 1.255 | 2 | 0.534 |
|  | Post-yield Work (mJ) | - | - | - | 6.093 | 2 | **overall: 0.048**  **FMR1^-/-^ vs. FMR1^+/+^: 0.027**  FMR1^+/-^ vs. FMR1^+/+^: 0.407 |
|  | Total Work (mJ) | 3.857 | 2 | **overall: 0.038**  **FMR1^-/-^ vs. FMR1^+/+^: 0.023**  FMR1^+/-^ vs. FMR1^+/+^: 0.219 | - | - | - |
|  | Yield Stress (MPa) | 0.108 | 2 | 0.898 | - | - | - |
|  | Ultimate Stress (MPa) | 0.334 | 2 | 0.720 | - | - | - |
|  | Modulus (GPa) | 0.428 | 2 | 0.658 | - | - | - |
|  | Resilience (MPa) | - | - | - | 0.708 | 2 | 0.702 |
|  | Toughness (MPa) | 1.770 | 2 | 0.196 | - | - | - |
| **9-month-old** | Yield Force (N) | 2.182 | 2 | 0.131 | - | - | - |
|  | Ultimate Force (N) | 5.263 | 2 | **overall: 0.011**  **FMR1^-/-^ vs. FMR1^+/+^: 0.023**  FMR1^+/-^ vs. FMR1^+/+^: 0.698 | - | - | - |
|  | Stiffness (N/mm) | 4.482 | 2 | **overall: 0.020**  **FMR1^-/-^ vs. FMR1^+/+^: 0.022**  FMR1^+/-^ vs. FMR1^+/+^: 0.907 | - | - | - |
|  | Work to Yield (mJ) | 1.346 | 2 | 0.276 | - | - | - |
|  | Post-yield Work (mJ) | 5.280 | 2 | **overall: 0.011**  **FMR1^-/-^ vs. FMR1^+/+^: 0.009**  FMR1^+/-^ vs. FMR1^+/+^: 0.616 | - | - | - |
|  | Total Work (mJ) | 6.792 | 2 | **overall: 0.004**  **FMR1^-/-^ vs. FMR1^+/+^: 0.003**  FMR1^+/-^ vs. FMR1^+/+^: 0.456 | - | - | - |
|  | Yield Stress (MPa) | - | - | - | 0.254 | 2 | 0.881 |
|  | Ultimate Stress (MPa) | 1.347 | 2 | 0.276 |  |  |  |
|  | Modulus (GPa) | - | - | **-** | 1.243 | 2 | 0.537 |
|  | Resilience (MPa) | 0.151 | 2 | 0.860 |  |  |  |
|  | Toughness (MPa) | 4.628 | 2 | **overall: 0.018**  **FMR1^-/-^ vs. FMR1^+/+^: 0.014**  FMR1^+/-^ vs. FMR1^+/+^: 0.631 | - | - | - |

**Supplementary Table 7. qPCR primer/probe sets used to analyze the genes included in Figures 5 and 6.**

| **Gene** | **Vendor** | **Primer ID** | | |
| --- | --- | --- | --- | --- |
| choB (Mrps2) | ABI | Mm00475529_m1 | | |
| FMR1 | ABI | Mm01339582_m1 | | |
| Cx43 | ABI | Mm00439105_m1 | | |
| Phex | ABI | Mm00448119_m1 | | |
|  |  | **Primer Sequence (forward)** | **Primer Sequence (reverse)** | **Universal Probe**  **(Millipore Sigma)** |
| Col1a1 | Roche | catgttcagctttgtggacct | gcagctgacttcagggatgt | #15 (cat. no. 04685148001) |
| osteocalcin | Roche | tgaggaccatctttctgctca | tggacatgaaggctttgtca | #71 (cat. no. 04688945001) |
| DMP1 | Roche | ggttttgaccttgtgggaaa | ttgggatgcgattcctctac | #106 (cat. no. 04692250001) |
| E11 | Roche | cagtgttgttctgggttttgg | tggggtcacaatatcatcttca | #95 (cat. no. 04692128001) |
| Sost | Roche | tcctgagaacaaccagacca | gcagctgtactcggacacatc | #16 (cat. no. 04686896001) |
| PTEN | Roche | aggcacaagaggccctagat | ctgactgggaattgtgactcc | #60 (cat. no. 04688589001) |
